# Adjunctive Effect of Systemic Antibiotics in Regenerative/Reconstructive Periodontal Surgery—A Systematic Review with Meta-Analysis

**DOI:** 10.3390/antibiotics11010008

**Published:** 2021-12-22

**Authors:** Luigi Nibali, Jacopo Buti, Luigi Barbato, Francesco Cairo, Filippo Graziani, Søren Jepsen

**Affiliations:** 1 Periodontology Unit, Centre for Host Microbiome Interactions, King’s College London, London SE1 9RT, UK; 2 Periodontology Department, UCL Eastman Dental Institute, University College London, London WC1E 6BT, UK; j.buti@ucl.ac.uk; 3 Research Unit in Periodontology and Periodontal Medicine, Department of Experimental and Clinical Medicine, University of Florence, 50121 Florence, Italy; luigi.barbato85@libero.it (L.B.); cairofrancesco@virgilio.it (F.C.); 4 Unit of Dentistry and Oral Surgery, Department of Surgical, Medical and Molecular Pathology and CriticalCare Medicine, University of Pisa, 56126 Pisa, Italy; filippo.graziani@med.unipi.it; 5 Department of Periodontology, Operative & Preventive Dentistry, University Hospital Bonn, 53127 Bonn, Germany; sjepsen@uni-bonn.de

**Keywords:** periodontitis, regeneration, antibiotic, infection, surgery

## Abstract

Background and Objective: Systemic antibiotics (AB) are often used in conjunction with regenerative/reconstructive periodontal surgery of intrabony defects and furcations; however, their potential benefits have not been systematically assessed. Materials and Methods: Data were retrieved from two recent systematic reviews (a total of 105 randomized clinical trials (RCTs) on clinical and radiographic outcomes in intrabony defects (ID) and molars with furcation involvement (FI) treated by surgical access with regenerative techniques. Pair-wise meta-analysis of RCTs with and without AB was performed. Meta-regressions from single-arm (subgroup) RCTs including study arms with or without adjunctive AB were also conducted. Results: No statistically significant benefits of systemic AB with regard to PPD, CAL and bone gain were detected in ID by pair-wise meta-analysis. Meta-regression revealed increased PPD reduction (−0.91 mm, 95% CI = −1.30; −0.51, *p* < 0.001), CAL gain (−0.92 mm, 95% CI = −1.32; −0.52, *p* < 0.001) and bone gain (−1.08 mm, 95% CI = −1.63; −0.53, *p* < 0.001) in ID but not in any of the outcomes in FI for arms treated with AB vs. study arms treated with no AB. No clear differences in adverse events were detected between AB and non-AB groups. Conclusion: There is only weak indirect evidence that AB may provide additional benefits in terms of clinical improvements in the regenerative/reconstructive periodontal surgery of intrabony defects and no evidence for a benefit in furcations. Until new data are gained and in the context of antibiotic stewardship, it may be questionable to justify the adjunctive use of systemic antibiotics.

## 1. Introduction

The use of systemic antimicrobials in periodontal therapy has been advocated for several decades, owing to the importance of microbes as triggers of the periodontal pathogenic process [1,2]. Several studies focused on the potential role of systemic antibiotics as adjuncts to mechanical non-surgical therapy, with the aim to improve pocket depth reduction and attachment level gain. A recent systematic review (SR) and meta-analysis concluded that the use of systemic antimicrobials as an adjunct to subgingival instrumentation results in statistically significant improvements in clinical outcomes. Adverse events, albeit usually not severe, are more frequent when antibiotics rather than placebo are used [3]. However, there is rising concern about the global emergence of antibiotic resistance [4,5]. and a recent S3-level clinical guideline called for a restricted use of adjunctive systemic antibiotics during step 2 of periodontal therapy [6]. 

The other common application of systemic antibiotics is during periodontal surgery, and particularly regenerative/reconstructive surgery. These procedures aim to stimulate the creation of new periodontal attachment with the use of various types of biomaterials, leading to a higher magnitude of clinical benefits in terms of pocket reduction and clinical attachment gain compared with flap surgery alone [7,8]. Antibiotics are prescribed with the objective to prevent post-operative infections (may reduce post-surgical complications) and to enhance clinical outcomes. However, this aspect has been rather under-researched compared with the use of antibiotics during the non-surgical phase [9,10,11]. Therefore, in the reality of clinical practice, antibiotics are often empirically prescribed in the case of regenerative periodontal surgery, with no clear evidence to support it [12,13]. However, to date, the potential benefits of systemic antibiotics as an adjunct to regenerative periodontal surgery have not been systematically appraised.

Therefore, we aimed to investigate the adjunctive benefit of systemic antimicrobials on clinical and radiographic outcomes in intrabony defects (ID) and molars with furcation involvement (FI) treated with periodontal regenerative/reconstructive surgery.

## 2. Materials and Methods

The PRISMA statement [14] was followed both in the planning [15] and reporting [16] of the review (Supplementary Material S1). Both original reviews providing data for the present review [7,8] had been registered on PROSPERO (ID references CRD42019124022 and CRD42019124466, respectively).

### 2.1. Focused Question

“In patients with periodontitis with ID or FI treated with regenerative surgery, what is the adjunctive benefit of systemic antimicrobials on clinical and radiographic outcomes in intrabony defects (ID) and molars with furcation involvement (FI) treated with periodontal regenerative/reconstructive surgery?”

### 2.2. Eligibility Criteria

The following PICOS method was the following:

- Population: patients with periodontitis with ID or FI treated with regenerative surgery.
- Intervention: adjunctive use of systemic antibiotics.
- Comparison: no use of systemic antibiotics.
- Outcomes: clinical attachment level (CAL) gain, probing pocket depth (PPD) reduction, bone gain.
- Studies: RCTs testing a regenerative technique at least in one arm and including at least 10 patients/arm and at least 1-year follow-up.

For this SR, we considered any type of regenerative surgery [guided tissue regeneration (GTR), enamel matrix derivative (EMD), bone filler or substitutes (BRG), growth factors (GF)] in patients with periodontal intra-bony defects ≥3 mm [7] or periodontitis-related furcation defects of any type (class II and III) [8]. Combination therapies were also considered.

### 2.3. Information Sources, Search and Study Selection

The searches from two recent SRs [7,8] were used as a starting point for this SR and the last update was performed on the 20th of November 2020. Briefly, all the studies included in the two SRs were reconsidered according to the PICO criteria for this review. Additionally, the list of the full texts excluded was also checked for additional titles. Then the searches strategies used in the previous reviews were updated. The new titles were screened, and full texts were evaluated according to the PICO criteria for this review (for additional information, see Supplementary Material S2).

### 2.4. Study Characteristics and Data Items

All papers included were screened for reporting the use of antimicrobials. When this was not clearly stated, senior authors of the respective papers were contacted to enquire whether antimicrobials were used or not as part of the study protocol.

The following outcomes from the included studies were registered:

- For IDs: CAL gain, PPD reduction and bone gain [7]
- For FIs: horizontal clinical attachment level (H-CAL) gain, vertical clinical attachment level (V-CAL), (PPD) reduction, horizontal bone level (HBL) gain [8]
- Information regarding Adverse Events (AE) were also considered.

### 2.5. Risk of Bias Analysis

The quality of the included studies was assessed using the Cochrane Collaboration’s Tool for assessing the risk of bias for RCTs [17]. The assessments of the risk of bias previously reported in Nibali et al. [7] and Jepsen et al. [8] were updated by adding the new papers included in this SR.

### 2.6. Summary Measures and Planned Method of Analysis

Data were grouped with respect to (a) regenerative biomaterials used (e.g., EMD, GTR-R, GTR-NR, BRG)—studies included EMD, GTR with resorbable and non-resorbable membranes, with or without bone replacement grafts; (b) antibiotics (AB+)/non-antibiotics (AB−) for each of the outcome measures and analyzed separately for IDs (CAL gain, PPD reduction and bone gain) and FIs (H-CAL gain, V-CAL gain, PPD reduction and H-BL gain). Standard pair-wise meta-analyses were conducted when studies directly comparing regenerative techniques with and without the adjunctive use of antibiotics were available. Random-effects models were used throughout. For continuous data, pooled outcomes were expressed as mean differences with their associated 95% confidence intervals (CI). The analyses were conducted using the generic inverse variance statistical method where the means and standard deviations (SD) were entered for each study arm. Statistical heterogeneity was assessed by calculation of the Q statistic. Analyses were performed using Review Manager (RevMan) [Computer program] (Version 5.3. Copenhagen: The Nordic Cochrane Centre, The Cochrane Collaboration, 2014). Furthermore, after grouping data with respect to the use or not of antibiotics (AB+/AB−), meta-analyses were performed, including individual arms of RCTs. The degree of heterogeneity between studies was quantified utilizing the I^2^ statistical test and subgroup and meta-regression analyses were performed to determine the effect of the adjunctive use of antibiotics (AB+ vs. AB−) and the type of antibiotics (penicillin vs. tetracycline) using specific software for meta-analysis (OpenMeta[Analyst]) (open-source software, Brown University of Public Health, Providence, RI, USA). The DerSimonian–Laird random-effects method was chosen. Results were presented as Forest plots with weighted mean values and 95% confidence intervals (CI). A *p* value < 0.05 was considered statistically significant.

## 3. Results

A total of 105 studies were included in this SR (see Supplementary Material S3 for a full list of included papers). Out of these 105 studies, 86 accounting for 150 treatment arms reported data on IDs, while 19 accounting for 35 treatment arms reported data on FIs (see flowchart diagram in Supplementary Material S4). Out of 105 included studies, in 75 the use of systemic antibiotics was reported after the surgery; in two studies, the use of systemic antibiotics was reported only the day of the surgery, while in 24 studies, systemic antibiotics were not used. Only four studies tested a systemic antibiotic vs. no antibiotic. The most used antibiotics were amoxicillin and doxycycline. Table 1 and Table 2 report details of antibiotic use in all included papers.

### 3.1. Risk of Bias

Out of the 105 included studies, 17 were rated at high risk of bias, 68 at unclear and only 20 at low risk of bias. Among the seven domains, the lack of blinding of the outcome assessor and incomplete outcome data were the most frequent sources of bias for both ID and FI studies (Supplementary Material S5).

### 3.2. Pair-Wise Meta-Analyses

**Figure 1:**
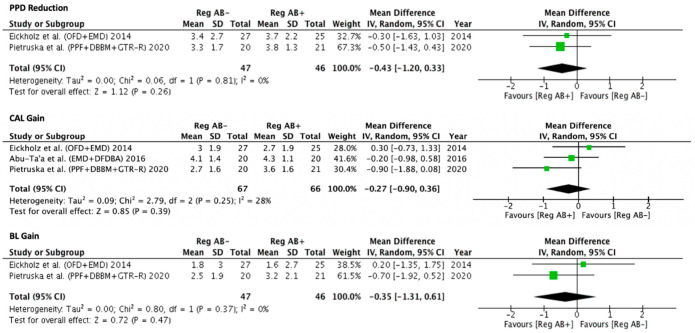
Forest plots of pair-wise meta-analyses for PPD reduction, CAL gain and bone gain for studies directly comparing regenerative techniques in intrabony defects with or without adjunctive antibiotics.

Intrabony defects: A total of four included studies tested adjunctive antibiotics in regenerative therapy of ID [11,18,19,20]. Among these, three could be included in meta-analysis [18,19,20], as the other did not report standard deviations. One of these studies used EMD + BRG (with or without amoxicillin) [18], the second EMD (with or without Doxycycline) ([19], and the third used GTR-R + BRG (with or without amoxicillin) ([20]. Pair-wise meta-analyses (Figure 1) showed lack-of statistically significant differences for antibiotic vs. no-antibiotic for PPD reduction (−0.43 mm, 95% CI = −1.20; 0.33, I^2^ = 0%), CAL gain (−0.27 mm, 95% CI= −0.90; 0.36, I^2^ = 28%) and bone gain (−0.35 mm, 95% CI= −1.31; 0.61, I^2^ = 0%).

Furcation involvement: No studies were available for pair-wise meta-analyses of AB vs. non-AB for FI.

### 3.3. Single-Arm Meta-Regression for Intrabony Defects

Single-arm meta-analyses for intrabony defects included between 39 and 82 antibiotic arms and between 15 and 32 non-antibiotic arms (depending on outcomes reported). Studies included EMD, GTR with resorbable and non-resorbable membranes, with or without bone replacement grafts. Antibiotics employed included penicillin, amoxicillin, amoxicillin + clavulanic acid, ampicillin, doxycycline, tetracycline, cephalosporin and clindamycin. The following outcomes were statistically significantly improved in AB vs. non-AB study arms (see summary in Table 3 and Forest Plots in Supplementary Material S6):

PPD reduction: 4.56 mm (95% CI = 4.33; 4.79, I^2^ = 95.73%) in AB arms vs. 3.64 mm (95% CI = 3.35; 3.94 mm, I^2^ = 88.98%) in non−AB arms (meta regression mean diff: −0.91, 95% CI = −1.30; −0.51, *p* < 0.001).

CAL gain: 3.71 mm (95% CI = 3.49; 3.94, I^2^ = 96.05%) in AB arms vs. 2.79 mm (95% CI = 2.33; 3.26 mm, I^2^ = 96.78%) in non−AB arms (meta regression mean diff: −0.92, 95% CI = −1.32; −0.52, *p* < 0.001).

Bone gain: 3.26 mm (95% CI = 2.99; 3.53, I^2^ = 93.71%) in AB arms vs. 2.17 mm (95% CI = 1.79; 2.56 mm, I^2^ = 85.06%) in non−AB arms (meta−regression mean diff: −1.08, 95% CI = −1.63; −0.53, *p* < 0.001).

Sub-analyses (meta-regression) by biomaterial in intrabony defects showed better clinical outcomes in studies using EMD in AB arms vs. non-AB arms for PPD reduction (meta-regression mean diff: −0.98, 95% CI = −1.65; −0.32, *p* = 0.004) and for CAL gain (meta-regression mean diff: −0.98, 95% CI = −1.77; −0.19, *p* = 0.015) (Table 3 and Supplementary Material S7); however, no statistically significant differences were estimated between AB and non-AB arms in studies using bone grafts and GTR with resorbable membranes for PPD reduction (meta-regression mean diff: −0.22, 95% CI = −0.94; 0.50, *p* = 0.553) and for CAL gain (meta−regression mean diff: −0.15, 95% CI = −0.91; 0.60, *p* = 0.688) (Table 3 and Supplementary Material S8).

Sub-analyses (meta-regression) by antibiotic type in intrabony defects showed no statistically significant differences in studies using antibiotics of the penicillin family (including 55 arms) vs. studies using tetracyclines (including 17 arms) for PPD reduction (meta-regression mean diff: −0.25, 95% CI = −0.79; 0.29, *p* = 0.360) and for CAL gain (meta-regression mean diff: −0.24, 95% CI = −0.73; 0.25, *p* = 0.338) (Table 3 and Supplementary Material S9).

### 3.4. Single-Arm Meta-Regression for Furcation Defects

**Figure 2:**
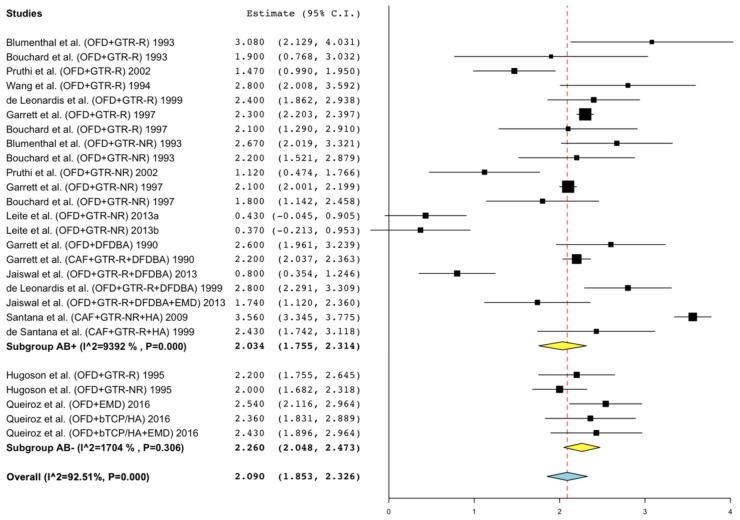
Forest plots of single-arm meta-analyses for PPD reduction for studies of regenerative techniques in furcation defects with or without adjunctive antibiotics.

Single-arm meta-analyses for furcation defects included between 9 and 21 antibiotic arms and between 2 and 7 non-antibiotic arms (depending on outcomes reported). Studies included a combination of EMD, GTR with resorbable and non-resorbable membranes and bone replacement grafts, alone or in combination. Antibiotics employed included penicillin, amoxicillin, amoxicillin + clavulanic acid, doxycycline and tetracycline. Non-statistically significant differences between AB and non-AB groups were estimated for:

PPD reduction: 2.03 mm (95% CI = 1.76; 2.31, I^2^ = 93.92%) in AB arms vs. 2.26 mm (95% CI = 2.05; 2.47, I^2^ = 17.04%) in non-AB arms (meta-regression mean diff: 0.27, 95% CI = −0.45; 0.98, *p* = 0.465) (Figure 2).

**Figure 3:**
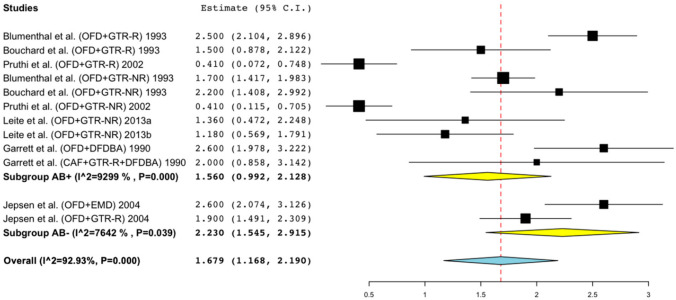
Forest plots of single-arm meta-analyses for H-BL gain for studies of regenerative techniques in furcation defects with or without adjunctive antibiotics.

H-BL gain: 1.56 mm (95% CI = 0.99; 2.13, I^2^ = 93.99%) in AB arms vs. 2.23 mm (95% CI = 1.55; 2.92, I^2^ = 76.42%) in non-AB arms (meta-regression mean diff: 0.69, 95% CI = −0.39; 1.78, *p* = 0.209) (Figure 3).

**Figure 4:**
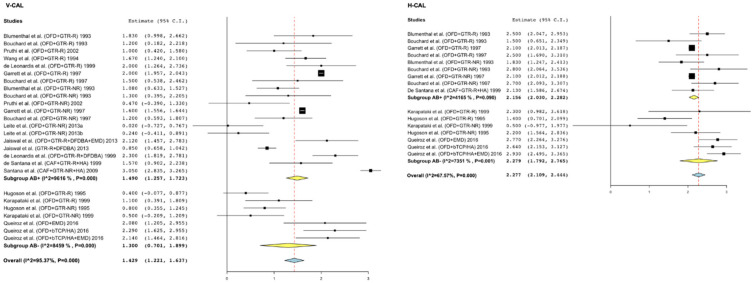
Forest plots of single-arm meta-analyses for V-CAL and H-CAL gain for studies of regenerative techniques in furcation defects with or without adjunctive antibiotics.

V-CAL gain: 1.50 mm (95% CI = 1.26; 1.72, I^2^ = 96.16%) in AB arms vs. 1.30 mm (95% CI = 0.70; 1.90, I^2^ = 84.59%) in non-AB arms (meta-regression mean diff: −0.16, 95% CI = 0.81; 0.50, *p* = 0.638) (Figure 4).

H-CAL gain: 2.16 mm (95%CI = 2.03; 2.28 mm, I^2^ = 41.65%) in AB arms vs. 2.28 mm (95%CI = 1.79; 2.77 mm, I^2^ = 73.51%) in non-AB arms (meta-regression mean diff: 0.16, 95%CI = 0.26; 0.57, *p* = 0.452) (Figure 4).

Sub-analysis by antibiotic type in furcation defects showed no statistically significant differences in studies using antibiotics of the penicillin family and studies using tetracyclines for PPD reduction (meta-regression mean diff: 0.34, 95% CI = 0.36; 1.05, *p* = 0.342) and for CAL gain (meta-regression mean diff: 0.31, 95% CI = −0.34; 0.96, *p* = 0.346) (Table 3 and Supplementary Material S10).

### 3.5. Adverse Events/Post-Operative Infections

No serious adverse events were reported in the studies included in this review.

#### 3.5.1. Pair-Wise Meta-Analysis Studies (ID)

Regarding the three studies included in the pair-wise meta-analysis of ID, Abu ta’a et al. [18] who had used EMD + BRG, stated that apart from erythema and swelling, no more severe postoperative complications such as suppuration, sloughing, perforation of the flap, and postoperative pain were reported in either of the groups. No information on adverse events/post-operative infection was provided by Eickholz et al. [19] who had employed EMD, whereas Pietruska et al. [20] that had used GTR-R + BRG reported that no signs of suppuration, extensive dehiscence or swelling were observed throughout the entire study period and that none of the patients reported intense pain, fever or discomfort.

#### 3.5.2. Single-Arm Meta-Regression Studies (ID)

Sixty-nine studies (55 AB+, 14 AB−) included in the single-arm meta-regression for ID clearly reported that there were no adverse events or post-operative infections during the healing period. In four studies (all AB+), no information on absence or presence of adverse events/post-operative infection was provided [21,22,23,24]. Another eight studies (AB+ and AB−) reported on unwanted postoperative healing events such as membrane exposure, slight inflammation, pain, swelling and bleeding but did not label these as adverse events or post-operative infections [25,26,27,28,29,30,31,32]. Only four ID studies specifically reported adverse events and/or postoperative infections. In the AB− arm of one study [11], a post-operative abscess occurred following the use of a resorbable membrane in one patient and was treated with antibiotics. Another infection of a site treated with a non-resorbable membrane was reported in a patient (AB+) after the surgery [33]. A pulp necrosis was observed in a study (AB+) using EMD [34]. In another study (AB−) using non-resorbable titanium-reinforced membranes, three patients were treated with antibiotics after membrane exposure without infection or abscesses [35]. In summary, adverse events/post-operative infections after regenerative treatment of IDs were rare.

#### 3.5.3. Single-Arm Meta-Regression Studies (FI)

For FI, in six of the studies (5 AB+, 1 AB−) included in the single-arm meta-regression, no information on post-operative adverse events or infections was reported at all. An additional seven studies (all AB+) explicitly stated that no post-operative infections or adverse tissue reactions had occurred. The remaining seven studies (4 AB+, 3 AB−) listed a few adverse healing events. Two studies (AB+) observed one abscess each following the use of a non-resorbable membrane [36,37]. Another parallel group study (AB+) reported 4 (out of 66) molars with abscess formation in the GTR-R group and 10 (out of 64) abscesses in the GTR-NR group [38]. In a split-mouth study (AB−), 13 (out of 38) patients had to receive antibiotics due to postoperative swelling, pain and suppuration, more frequently associated with the teeth that had been treated with GTR-NR [39]. In another split-mouth study (AB−) in 4 (out of 11) patients, antibiotics were prescribed because of postoperative pain, swelling and suppuration following GTR-R and GTR-NR [40]. In the third (AB−) study [41], antibiotics were given due to signs of post-operative infection in 8 (out of 45) teeth following GTR-R and in 2 (out of 45) teeth following EMD treatment.

In summary, post-operative infections after regenerative treatment of FI occurred infrequently in studies and treatment arms with and without a post-operative antibiotic protocol and appeared to be mostly related to the use of non-resorbable membranes.

The exposure of the membrane, biomaterial and the lack of primary closure were the most common complications reported in both studies with and without antibiotic treatment. The incidence, the assessment and the influence of these complications on the regenerative outcomes vary across the studies; thus, a meta-analysis was not considered appropriate.

## 4. Discussion

This systematic review provides weak evidence that adjunctive systemic antibiotics could lead to slight additional clinical and radiographic benefits as part of the surgical regenerative periodontal treatment of intrabony defects. No such evidence could be obtained with regard to the regenerative treatment of furcation defects.

This systematic review addresses a very important question. The use of antibiotics in periodontology is still controversial. Studies have shown that the development of antibiotic resistance in periodontal pathogens exists [42,43,44]. A high proportion of patients with resistant pathogens was described by Rams et al. [42], where 74% of patients were reported to have subgingival periodontal pathogens resistant to at least one of the antibiotics commonly used in clinical practice. Recently, periodontal pathogens collected from almost 8000 patients with periodontitis were shown to be non-susceptible to at least one of the antibiotics tested in about two-thirds of the patients [44] and the data further revealed a trend towards decreasing susceptibility profiles. While a targeted antimicrobial approach, based on microbial analysis, may be considered in the context of step 2 of periodontal therapy [6], aiming at the reduction in the causative agents (subgingival bacterial biofilm on the root surface), this may not be appropriate for step 3 therapy, where the aim of adjunctive antibiotics is prevention/prophylaxis of wound/biomaterial infection. In the era of increasing antimicrobial resistance, public health needs demand a reduction in the use of unnecessary antibiotic usage through antibiotic stewardship [45]. Although adjunctive systemic antibiotics have been shown to lead to enhanced clinical and radiographic outcomes following non-surgical periodontal therapy [3], a recent S3-level clinical guideline calls for a rather restrictive use in the non-surgical phase [6]; however, no evidence-based guidelines deal with the adjunctive prophylactic use of antibiotics in conjunction with periodontal surgery [12,13,46,47]. Despite their common use in practice, it has been unclear if their use in this scenario is justifiable. The data produced by this systematic review can contribute to the debate about whether systemic antibiotics should be prescribed concomitantly to regenerative periodontal surgery.

Traditional meta-analysis could only be performed for data derived by two RCTs in ID and revealed the absence of adjunctive clinical benefits associated with the use of systemic AB. This is in agreement with some other studies on this topic, which could not be included in the meta-analysis due to the review’s inclusion criteria or lack of reported data [10,11]; however, meta-regression of single-arm studies showed a higher magnitude of improvements of most investigated clinical and radiographic measures in ID. In particular, PPD and CAL were improved more in ‘antibiotic’ arms compared with ‘non-antibiotic arms’, both with differences just short of 1 mm. The radiographic bone gain was just over 1 mm higher in ‘antibiotic arms’ compared with ‘non-antibiotic arms’. It could be speculated that this difference may be due to the reduced risk of infection during the healing process, which may impair the regenerative process, especially at early stages. Although the potential benefit of prescribing systemic antibiotics is often sought when applying barrier membranes and/or bone substitutes, the data analyzed in this review pointed towards an increased benefit in studies using EMD, rather than in studies using GTR with resorbable membranes and bone grafts. Even if modern literature has extensively demonstrated that papillary preservation flaps are mandatory for effective regenerative procedures [7], systemic antibiotics may increase the protection of regeneration sites against infection sources in the oral cavity in conjunction with primary wound closure. In this context, it is interesting that the additional clinical and radiographic improvements appeared only in intrabony studies but not for regenerative/reconstructive surgery of furcation defects. It may be speculated that this could be due to the challenge of primary flap closure for IDs, mostly located interdentally. In contrast, flap access and adequate wound closure are less demanding in sites with FI, in studies mostly located buccally. It should be added that although adjunctive antibiotic therapy may be recommended in immunocompromised patients or individuals with high infection risk, such patients are usually excluded from studies on regenerative periodontal treatment, and often from periodontal surgery in clinical practice.

No serious adverse events were reported in any of the included studies. Thus, regenerative treatments at both ID and FI, with or without systemic antibiotics, could be considered safe procedures. In 86 studies on ID, only two patients with post-operative infections, one in an AB+ and another in an AB− group, were reported. This is in contrast to the 19 studies on FI, where 16 out of 157 patients (10%) in AB+ study arms of four studies experienced post-operative abscesses, whereas 25 out of 139 patients (18%) in AB− study arms (three studies) experienced postoperative pain, swelling and suppuration, that were treated by antibiotics. Thus, very little data are available on post-operative infections. Moreover, they occurred in study arms with and without post-operative administration of systemic antibiotics and, therefore, it is difficult to assess the influence of the use of AB. Interestingly, almost all the infections occurred in sites with a barrier membrane. In one RCT, 100% of the defects treated with a resorbable membrane showed at least a complication compared to 6% of EMD-treated defects [29]. Barrier membrane exposure was the most common complication reported in the studies included in this review. These data support the hypothesis that the use of barrier membranes could increase the risk of infection during the early healing stages. In this context, the protective effects of adjunctive AB on membrane exposure remain unclear. A recent RCT tested the effect of an AB on regeneration by means of DBBM and a resorbable membrane. Membrane exposure was reported in seven control patients not receiving AB and six test patients receiving AB. No signs of swelling or suppuration were reported in any case [20]. Interestingly, another randomized controlled trial showed that patients’ subjective perception of postoperative discomfort was significantly smaller in the group receiving antibiotics compared with the placebo group [18].

Sub-analysis on the type of adjunctive antibiotics used showed no differences in PD and CAL gain in ID when antibiotics of the penicillin or tetracycline families were used. This needs to be interpreted cautiously due to the lack of direct comparisons.

The strength of this review is that it is the first, to our knowledge, to combine data on regenerative therapy of intrabony defects and furcations to assess the potential effect of adjunctive antibiotics. Furthermore, a very large number of studies were included. Limitations include high heterogeneity of most of the analyses and the fact that less than 20% of included papers were judged to have a low risk of bias, with items such as lack of blinding of the outcome assessor and incomplete outcome data considered as the most frequent sources of bias. The heterogeneity is likely due to the high variability in regenerative procedures and antibiotic protocols among selected trials. This heterogeneity may hinder the final selection of the proper approach in infection prevention. It should also be stressed that, due to the nature of the meta-regression, we cannot conclude that the observed differences between antibiotic and non-antibiotic arms were entirely due to the use of antibiotics.

Thus, future research in this area may be necessary by well-designed RCTs to prove or disprove the benefits of adjunctive systemic antibiotics during regenerative/reconstructive surgery. It will be important to standardize protocols treatment protocols, for example, by including in future RCTs testing adjunctive systemic antibiotics in step 3 of periodontal therapy only patients who received or did not receive such antibiotics in step 2.

However, recent reviews and surveys have emphasized the low incidence of infection in periodontal surgery irrespective of the use of systemic antibiotics, have suggested not to use them in the perioperative period to prevent infection [12] and have demanded the development of guidelines [13,47].

## 5. Conclusions

At present, based on the results of the present systematic review, with the available weak evidence for additional benefits attributable to systemic antibiotics in the regenerative/reconstructive periodontal surgery of intrabony defects and no evidence for their additional benefit in furcation defects, it appears questionable to justify their adjunctive use. 

## Figures and Tables

**Table 1:** Characteristics of the treatment arms from included studies treating intra-bony defects (ID).

Author/Treatment	Country	N° Patients	N° Defects	Antibiotic Regimen	CAL Gain	PPD Reduction	Bone Gain
**EMD**							
Silvestri et al. (OFD + EMD) 2000	Italy	10	10	Amoxicillin 875 + clavulanic 125 b.i.d. for 7 days	4.5 ± 1.6	4.8 ± 1.6	-
Heijl et al. (OFD + EMD) 1997	Sweden	26	27	Doxycycline 200 mg day one, then 100 mg q.d. for 3 weeks	2.2 ± 1.1	3.1 ± 1	2.6 ± 1.7
Okuda et al. (OFD + EMD) 2000	Japan	16	18	Cafaclor 750 mg q.d. 5 days	1.72 ± 1.07	3 ± 0.97	-
Crea et al. (OFD + EMD) 2008	Italy	19	19	Amoxicillin 500 mg b.i.d. for 7 days	2.4 ± 1.1	3.1 ± 1.4	2.7 ± 1
Iorio-Siciliano et al. (OFD + EMD) 2011	Italy	20	20	No antibiotic	2.4 ± 2.2	2.9 ± 2.1	-
Silvestri et al. (OFD + EMD) 2003	Italy	49	49	Amoxicillin 875 + clavulanic 125 b.i.d. for 7 days	4.1 ± 1.8	5.3 ± 1.9	-
Pontoriero et al. (OFD + EMD) 1999	Sweden	10	10	3G amoxicillin 1 h before surgery	2.9 *	4.4 *	-
Sanz et al. (OFD + EMD) 2004	Spain	35	35	No antibiotics	3.1 ± 1.8	3.8 ± 1.5	-
Minabe et al. (OFD + EMD) 2002	Japan	-	22	Cefaclor 750 mg q.d. for 4 days	2.6 ± 1	3.8 ± 0.9	-
Guida et al. (OFD + EMD) 2007	Italy	14	14	Amoxicillin 875 + clavulanic 125 b.i.d. for 6 days	4.6 ± 1.3	5.6 ± 1.7	4.3 ± 2.4
Yilmaz et al. (OFD + EMD) 2010	Turkey	20	20	Amoxicillin 500 mg t.i.d. for 7 days	3.4 ± 0.8	4.6 ± 0.4	2.8 ± 0.8
Losada et al. (OFD + EMD) 2016	Spain	23	23	Amoxicillin 750 or Clindamycin 300 mg t.i.d. for 7 days	2.65 ± 2.18	3.3 ± 1.89	2.6 ± 2.03
Meyle et al. (OFD + EMD) 2011	Germany	35	35	No antibiotics	1.93 ± 1.7	2.9 ± 1.8	2.81 ± 1.6
Pietruska et al. (OFD + EMD) 2011	Poland	12	12	Amoxicillin 500 mg t.i.d. for 7 days	§	§	-
Sculean et al. (OFD + EMD) 2005b	Netherlands/Poland	15	15	No antibiotics	3.9 ± 1.8	4.5 ± 2	-
Zucchelli et al. (OFD + EMD) 2003	Italy	30	30	Amoxicillin 875 + clavulanic 125 q.d. for 6 days	4.9 ± 1	5.8 ± 0.8	4.3 ± 1.5
Sipos et al. (OFD + EMD) 2005	Netherlands	11	12	No antibiotics	1.28 ± 2.04	2.86 ± 0.75	1.63 ± 1.21
Leknes et al. (OFD + EMD) 2009	Norway	13	13	No antibiotics	0.6 ± 1	2.5 ±1.9	-
Al Machot et al. (OFD + EMD) 2014	Germany	19	19	No antibiotics	1.4 ± 1.8	2.6 ± 1.8	1.6 ± 1.2
Eickholz et al. (OFD + EMD) 2014a	Germany	28	28	Doxycycline 200 mg q.d. for 7 days	2.74 ± 1.89	3.69 ± 2.23	-
Eickholz et al. (OFD + EMD) 2014b	Germany	29	29	No antibiotics	2.95 ± 1.92	3.4 ± 1.73	-
De Leonardis et al. (PPF + EMD) 2013	Italy	34	34	Amoxicillin 2 g q.d for 6 days	2.95 ± 0.74	3.76 ± 0.74	2.61 ± 0.49
Fickl et al. (PPF + EMD) 2009	Germany	19	35	No antibiotics	3.7 ± 0.4	4.2 ± 0.3	2.5 ± 0.4
Francetti et al. (PPF + EMD) 2004	Italy	11	11	Amoxicillin 875 mg + clavulanic 125 mg b.i.d. for 5 days	4.29 ± 1.38	4.86 ± 1.95	3.44 ± 1.18
Tonetti et al. (PPF + EMD) 2002	UK	83	83	No antibiotics	3.1 ± 1.5	3.9 ± 1.7	-
Zucchelli et al. (PPF + EMD) 2002	Italy	30	30	Amoxicillin 875 mg + clavulanic 125 mg q.d for 7 days	4.2 ± 0.9	5.1 ± 0.7	-
Francetti et al. (PPF + EMD) 2005	Italy	64	82	Amoxicillin 875 mg + clavulanic 125 mg b.i.d. for 5 days	3.51 *	4.02 *	-
Wachtel et al. (PPF + EMD) 2003	Germany	11	26	No antibiotics	3.6 ± 1.6	3.9 ± 1.4	-
Grusovin et al. (PPF + EMD) 2009	UK	15	15	No antibiotics	3.4 ± 1.1	4.2 ± 1.6	2.5 ± 1.2
Rosing et al. (PPF + EMD) 2005	Norway	14	14	Penicillin 500 mg t.i.d for 5 days	§	§	§
Bokan et al. (PPF + EMD) 2006	Germany	19	19	Doxycycline 100 mg q.d. for 7 days	3.7 ± 1	3.9 ± 1.3	-
Aslan et al. (PPF + EMD) 2020	Turkey	15	15	Doxycycline 100 mg b.i.d. for 7 days	6.3 ± 2.5	6.5 ± 2.65	-
Mazzonetto et al. (PPF + EMD) 2020	Brazil	20	20	No antibiotics	2.4 ± 1	2.3 ± 1.2	1.24 ± 1.14
**EMD + graft**							
Paolantonio et al. (PPF + EMD + AB) 2020	Italy	22	22	Amoxicillin 875 mg + clavulanic 125 mg b.i.d. for 6 days	3.29 ± 0.85	3.96 ± 1.04	2.67 ± 1.06
Guida et al. (OFD + EMD + AB) 2007	Italy	13	14	Amoxicillin 875 + clavulanic 125 b.i.d. for 6 days	4.9 ± 1.8	5.1 ± 1.7	4.3 ± 1.3
Yilmaz et al. (OFD + EMD + AB) 2010	Turkey	20	20	Amoxicillin 500 mg t.i.d. for 7 days	4.2 ± 1.1	5.6 ± 0.9	3.9 ± 1
Sculean et al. (OFD + EMD + BG) 2005b	Netherlands/Poland	15	15	No antibiotics	3.2 ± 1.7	4.2 ± 1.4	-
Sculean et al. (OFD + EMD + BG) 2002b	Germany	14	14	Amoxicillin 500 mg t.i.d. for 7 days	§	§	-
Ghezzi et al. (PPF + EMD + DBBM) 2016	Italy	10	10	Amoxicillin 875 + clavulanic 125 b.i.d. for 7 days	4.4 ± 1.17	4.9 ± 1.2	-
Sculean et al. (OFD + EMD + DBBM) 2002a	Germany	12	12	Amoxicillin 500 mg t.i.d. for 7 days	4.7 ± 1.9	5.7 ± 1.5	-
Iorio-Siciliano et al. (OFD + EMD + DBBM) 2014	Italy	20	20	No antibiotics	3.8 ± 1.6	4.6 ± 1.9	-
Döri et al. (OFD + EMD + DBBM) 2005	Hungary	12	12	Amoxicillin 500 mg t.i.d. for 7 days	4.3 ± 0.8	4.8 ± 0.9	-
Döri et al. (OFD + EMD + DBBM) 2008b	Hungary	13	13	Amoxicillin 500 mg t.i.d. for 7 days	5 ± 0.9	5.9 ± 1.3	-
Zucchelli et al. (OFD + EMD + DBBM) 2003	Italy	30	30	Amoxicillin 875 + clavulanic 125 q.d. for 6 days	5.8 ± 1.1	6.2 ± 0.4	5.3 ± 1.1
Aspriello et al. (OFD + EMD + DFDBA) 2011	Italy	28	28	Ceftibuten 400 mg q.d. for 6 day	4 *	5 *	4 *
Abu-ta et al. (OFD + EMD + DFDBA) 2016	Palestine	20	20	Amoxicillin 1 g pre-operative and 2 g q.d. for two days	4.3 ± 1.1	3.4 ± 1.2	-
Abu-ta et al. (OFD + EMD + DFDBA) 2016	Palestine	20	20	No antibiotics	4.1 ± 1.4	3.7 ± 1.3	-
Döri et al. (OFD + EMD + bTCP) 2005	Hungary	12	12	Amoxicillin 500 mg t.i.d. for 7 days	4.1 ± 0.8	4.6 ± 0.8	-
Losada et al. (OFD + EMD + HA/bTCP) 2016	Spain	21	21	Amoxicillin 750 or Clindamycin 300 mg t.i.d. for 7 days	2.38 ± 2.17	3.14 ± 1.95	2.71 ± 1.79
Meyle et al. (OFD + EMD + HA/bTCP) 2011	Germany	38	38	No antibiotics	1.69 ± 2.1	2.8 ± 2.1	2.65 ± 1.9
Pietruska et al. (OFD + EMD + HA/bTCP) 2011	Pietruska	12	12	Amoxicillin 500 mg t.i.d. for 7 days	§	§	-
Bokan et al. (PPF + EMD + bTCP) 2006	Germany	19	19	Doxycycline 100 mg q.d. for 7 days	4 ± 1	4.1 ± 1.2	-
Lee et al. (OFD + EMD + DPBM) 2020	Korea	20	20	Amoxicillin 500 mg t.i.d. for 5 days	§	§	-
**GTR-NR**							
Silvestri et al. (OFD + GTR-NR) 2000	Italy	10	10	Amoxicillin 875 + clavulanic 125 b.i.d. for 7 days	4.8 ± 1.6	5.9 ± 1.1	-
Mora et al. (OFD + GTR-NR) 1996	France	10	10	Tetracycline 500 mg q.d. 8 days	3.85 ± 0.9	5.35 ± 1.1	2.95 ± 1.3
Crea et al. (OFD + GTR-NR) 2008	Italy	20	20	Amoxicillin 500 mg b.i.d. for 7 days	2 ± 1.1	3.2 ± 1.1	2.7 ± 1.2
Iorio-Siciliano et al. (OFD + GTR-NR) 2011	Italy	20	20	No antibiotics	4.1 ± 1.4	5.5 ± 1	-
Silvestri et al. (OFD + GTR-NR) 2003	Italy	49	49	Amoxicillin 875 + clavulanic 125 b.i.d. for 7 days	4.3 ± 1.9	5.6 ± 1.5	-
Pontoriero et al. (OFD + GTR-NR) 1999	Sweden	10	10	3G amoxicillin 1 h before surgery	2.9 *	4.7 *	-
Christgau et al. (OFD + GTR-NR) 1997	Germany	10	10	Doxycycline 100 mg q.d. for 10 days	3.7 ± 3	3.9 ± 2.3	-
Zybutz et al. (OFD + GTR-NR) 2000	USA	14	14	The day of surgery. Molecule and dosage not specified	2.4 ± 0.8	3.1 ± 1.2	2.2 ± 1.7
Zucchelli et al. (PPF + GTR-NR) 2002	Italy	30	30	Amoxicillin 875 + clavulanic 125 q.d. for 7 days	4.9 ± 1.6	6.5 ± 1.6	-
**GTR-R**							
Mayfield et al. (OFD + GTR-R) 1998	Sweden	20	20	No antibiotics	1.5 ± 1.9	2.9 ± 1.8	0.6 ± 1.2
Paolantonio et al. (OFD + GTR-R) 2008	Italy	17	17	Ampicillin 1 g b.i.d. for 7 days	3.1 *	5.2 *	2.4 *
Loos et al. (OFD + GTR-R) 2002	Netherlands	13	13	Amoxicillin 375 mg and metronidazole 250 mg t.i.d. 8 days	1.3 *	-	-
Loos et al. (OFD + GTR-R) 2002	Netherlands	12	12	No antibiotics	1.5 *	-	-
Blumenthal et al. (OFD + GTR-R) 1990	USA	15	15	Tetracycline 250 mg q.d. for 10 days	1.17 ± 0.1	1.99 ± 0.3	1.83 ± 0.2
Pontoriero et al. (OFD + GTR-R) 1999	Sweden	10	10	3G amoxicillin 1 h before surgery	3 *	4.1 *	-
Pontoriero et al. (OFD + GTR-R) 1999	Sweden	10	10	3G amoxicillin 1 h before surgery	3.4 *	4.8 *	-
Sanz et al. (OFD + GTR-R) 2004	Spain	32	32	No antibiotics	2.5 ± 1.9	3.3 ± 1.5	-
Minabe et al. (OFD + GTR-R) 2002	Japan	-	24	Cefaclor 750 mg q.d. for 4 days	2.8 ± 0.9	3.7 ± 1.2	-
Christgau et al. (OFD + GTR-R) 1997	Germany	10	10	Doxycycline 100 mg q.d. for 10 days	3.8 ± 1.9	4 ± 1.4	-
Zybutz et al. (OFD + GTR-R) 2000	USA	15	15	The day of surgery. Molecule and dosage not specified	2.4 ± 1.9	3.3 ± 2.1	2.4 ± 3.7
Trejo et al. (OFD + GTR-R) 2000	USA	14	14	Doxycycline 100 mg q.d. for 10 days	3.27 ± 1.1	4.12 ± 0.84	5.35 ± 2.91
Paolantonio et al. (OFD + GTR-R) 2002	Italy	17	17	Ampicillin 1 g b.i.d. for 7 days	4 ± 1.27	5.58 ± 1	3.82 ± 1.28
Mengel et al. (OFD + GTR-R) 2003	Germany	-	22	No antibiotics	3.4 ± 2.3	4 ± 2.1	-
Tonetti et al. (PPF + GTR-R) 1998	Switzerland	69	69	Doxycycline 100 mg b.i.d. for 7 days	3.04 ± 1.64	4.03 ± 1.81	-
Cortellini et al. (PPF + GTR-R) 2001	Italy	55	55	Doxycycline 200 mg q.d. for 7 days	3.5 ± 2.1	4.4 ± 2.4	-
Stravopolous et al. (PPF + GTR-R) 2003	Denmark	14	14	Amoxicillin 750 mg + metronidazole 250 mg q.d. for 5 days	2.9 *	3.9 *	3.1 *
**Graft + GTR-R**							
Sculean et al. (OFD + DBBM + GTR-R) 2003	Germany	14	14	Amoxicillin 500 mg t.i.d. for 7 days	4 ± 1.3	5.3 ± 1.6	-
Sculean et al. (OFD + DBBM + GTR-R) 2005a	Netherlands	16	16	No antibiotics	4.1 ± 0.9	5.4 ± 0.9	-
Tonetti et al. (OFD + DBBM + GTR-R) 2004	UK	61	61	Doxycycline 200 mg q.d. for 7 days	3.3 ± 1.7	3.7 ± 1.8	-
Iorio-Siciliano et al. (OFD + DBBM + GTR-R) 2014	Italy	20	20	No antibiotics	3.7 ± 1.2	4.4 ± 1.7	-
Döri et al. (OFD + DBBM + GTR-R) 2007b	Hungary	12	12	Amoxicillin 500 mg t.i.d. for 7 days	4.6 ± 0.8	5.7 ± 1.2	-
Paolantonio et al. (OFD + DBBM + GTR-R) 2002	Italy	17	17	Ampicillin 1 g b.i.d. for 7 days	5.05 ± 1.56	5.76 ± 1.6	5.23 ± 1.3
Stravopolous et al. (PPF + DBBM + GTR-R) 2003	Denmark	15	15	Amoxicillin 750 mg + metronidazole 250 mg q.d. for 5 days	2.5 *	3.8 *	2.8 *
Ghezzi et al. (OFD + DBBM + GTR-R) 2016	Italy	10	10	Amoxicillin 875 + clavulanic 125 b.i.d. for 7 days	4 ± 1.82	4.7 ± 2.36	-
Pietruska et al. (PPF + DBBM + GTR-R) 2020	Poland	21	21	Amoxicillin 2 g q.d. for 7 days	3.6 ± 1.6	3.8 ± 1.3	3.2 ± 2.1
Pietruska et al. (PPF + DBBM + GTR-R) 2020	Poland	20	20	No antibiotics	2.7 ± 1.6	3.3 ± 1.7	2.5 ± 1.9
Kim et al. (OFD + DFDBA + CS barrier) 1998	Korea	13	13	Tetracycline 250 mg q.d. for 7 days	2.9 ± 0.8	4.3 ± 0.5	2.9 ± 1.4
Trejo et al. (OFD + DFDBA + GTR-R) 2000	USA	16	16	Doxycycline 100 mg q.d. for 10 days	2.29 ± 0.61	3.37 ± 1.16	4.73 ± 1.18
Christgau et al. (OFD + bTCP + GTR-R) 2006	Germany	25	25	Doxycycline 100 mg q.d. for 19 days	5.2 ± 1.6	6 ± 1.1	-
Orsini et al. (OFD + AB + CSM) 2008	Italy/Spain	12	12	Antibiotic for 1 week. The molecule and dosage were not specified	2.6 ± 1.2	3.3 ± 1.6	-
Orsini et al. (OFD + AB + GTR-R) 2008	Italy/Spain	12	12	Antibiotic for 1 week. The molecule and dosage were not specified	2.4 ± 1.1	4.2 ± 1.2	-
Cetinkaya et al. (OFD + BG + GTR-R) 2014	Turkey	11	11	No Antibiotics	2.64 ± 1.12	3.45 ± 0.93	3 ± 1.48
Yamamiya et al. (OFD + HA + HCPC) 2008	Japan	15	15	Cefaclor 750 mg q.d. for 5 days	2.7 ± 1.3	4.3 ± 1.1	3.2 ± 1.1
**Graft + GTR-NR**							
Nygaard-Østby et al. (OFD + AB + GTR-NR) 2010	Norway	13	13	Amoxicillin 500 mg b.i.d. for 10 days	3.8 ± 0.5	4.2 ± 0.5	3.9 ± 0.8
Döri et al. (OFD + bTCP + GTR-NR) 2008a	Hungary	14	14	Amoxicillin 500 mg t.i.d. for 7 days	3.9 ± 0.2	5.4 ± 0.7	-
Döri et al. (OFD + DBBM + GTR-NR) 2007a	Hungary	15	15	Amoxicillin 500 mg t.i.d. for 7 days	4.6 ± 1.1	5.5 ± 1.7	-
**Graft**							
Nygaard-Østby et al. (OFD + AB) 2010	Norway	13	13	Amoxicillin 500 mg b.i.d. for 10 days	2.2 ± 0.7	2.7 ± 0.5	1.3 ± 0.9
Leknes et al. (OFD + BG) 2009	Norway	13	13	No Antibiotics	1.2 ± 0.2	2.6 ± 1.1	-
Mengel et al. (OFD + BG) 2003	Germany	-	20	No Antibiotics	2.8 ± 1.8	3.8 ± 1.9	-
Sculean et al. (OFD + BG) 2002b	Germany	14	14	Amoxicillin 500 mg t.i.d. for 7 days	§	§	-
Yassibag-Berkman et al. (OFD + bTCP) 2007	Turkey	-	10	Amoxicillin 875 + clavulanic 125 b.i.d. for 5 days	2.5 *	4.1 *	-
Pietruska et al. (OFD + HA) 2012	Poland	15	15	Amoxicillin 1 g b.i.d. for 7 days	2 ± 2.7	2.9 ± 2.5	1.9 ± 1.5
Al Machot et al. (OFD + HA) 2014	Germany	19	19	No Antibiotics	2.1 ± 1.6	3.2 ± 1.8	1.6 ± 1.2
Kasaj et al. (OFD + HA/p-15) 2008	Germany	13	13	No antibiotics	3.9 ± 1.7	4.3 ± 1.3	-
Okuda et al. (OFD + HA + saline) 2005	Japan	35	35	Cefaclor 750 mg q.d. for 5 days	2 ± 1.2	3.7 ± 2	2.7 ± 1.6
De Leonardis et al. (PPF + HA/bTCP) 2013	Italy	34	34	Amoxicillin 2 g q.d. for 6 days	3.63 ± 0.91	4.25 ± 0.63	3.35 ± 0.8
Scabbia et al. (PPF + HA/collagen/chondroitin sulfate) 2004	Italy	13	13	No Antibiotics	2.9 ± 1.9	4.2 ± 2.1	2.5 ± 1.4
Slotte et al. (OFD + DBBM) 2012	Sweden	16	16	Phenoxymethylpenicillin 2 g or clindamycin 300 mg b.i.d. for 7 days	2.3 ± 0.8	3.2 ± 0.7	3.4 ± 2.3
Sculean et al. (OFD + DBBM) 2002a	Germany	12	12	Amoxicillin 500 mg t.i.d. for 7 days	4.9 ± 2.1	6.5 ± 2	-
Döri et al. (OFD + DBBM) 2009	Hungary	15	15	Amoxicillin 500 mg t.i.d. for 7 days	4.7 ± 1.6	5.3 ± 1.7	-
Qiao et al. (OFD + DBBM) 2016	China	-	16	Amoxicillin 500 mg t.i.d. for 7 days	2.4 ± 1.1	3 ± 1.6	2.1 ± 1.5
Scabbia et al. (PPF + DBBM) 2004	Italy	11	11	No Antibiotics	4 ± 2.4	4.4 ± 2.3	3.1 ± 1.8
Agarwal et al. (OFD + DFDBA) 2014	India	24	24	Amoxicillin 500 mg t.i.d. for 7 days	2.4 ± 0.61	3.65 ± 0.52	2.37 ± 0.47
Piemontese et al. (OFD + DFDBA) 2008	Italy	30	30	Ceftibuten 400 mg q.d. for 5 days	2.4 ± 2.2	3.5 ± 1.9	2.6 ± 1.8
Agarwal et al. (OFD + DFDBA) 2015	India	30	30	Amoxicillin 500 mg t.i.d. for 7 days	2.61 ± 0.68	3.6 ± 0.51	2.49 ± 0.64
Aspriello et al. (OFD + DFDBA) 2011	Italy	28	28	Ceftibuten 400 mg q.d. for 6 days	3.25 *	4 *	3.5 *
Blumenthal et al. (OFD + AAA) 1990	USA	-	14	Tetracycline 250 mg q.d. for 10 days	1.43 ± 0.1	2.03 ± 0.1	2.06 ± 0.1
Shirakata et al. (OFD + CPC) 2008	Japan	15	15	The molecule and dosage were not specified.	2.3 ± 1	3.4 ± 1.2	1.2 ± 0.8
Paolantonio et al. (OFD + CS) 2008	Italy	17	17	Ampicillin 1 g b.i.d. for 7 days	2.7 *	4.4 *	2.3 *
Lee et al. (OFD + DPBM) 2020	Korea	22	22	Amoxicillin 500 mg t.i.d. for 5 days	§	§	-
Minenna et al. (OFD + PLA/PGA) 2005	Italy	16	16	No antibiotics	3.6 ± 1.5	4.6 ± 2	-
**PRP/PRF**							
Thorat et al. (OFD + PRF) 2017	India	15	15	Amoxicillin 500 mg +metronidazole 400 mg q.d. for 7 days	4 ± 0.63	4 ± 0.63	-
Patel et al. (OFD + PRF) 2017	India	13	13	Amoxicillin 500 mg t.i.d. for 7 days	3.7 ± 0.67	4.2 ± 1.69	-
**Graft + PRP/PRF**							
Döri et al. (OFD + DBBM + PRP) 2009	Hungary	15	15	Amoxicillin 500 mg t.i.d. for 7 days	4.6 ± 1.7	5.2 ± 1.6	-
Qiao et al. (OFD + DBBM + PRP(CGF)) 2016	China	-	15	Amoxicillin 500 mg t.i.d. for 7 days	3.7 ± 1.3	4.2 ± 1.3	3.3 ± 1.5
Agarwal et al. (OFD + DFDBA + PRF) 2015	India	30	30	Amoxicillin 500 mg t.i.d. for 7 days	3.73 ± 0.74	4.15 ± 0.84	3.5 ± 0.67
Agarwal et al. (OFD + DFDBA + PRP) 2014	India	24	24	Amoxicillin 500 mg t.i.d. for 7 days	3.15 ± 0.5	3.64 ± 0.63	3.02 ± 0.5
Piemontese et al. (OFD + DFDBA + PRP) 2008	Italy	30	30	Ceftibuten 400 mg q.d. for 5 days	3.6 ± 1.8	4.6 ± 1.3	3.3 ± 1.5
Okuda et al. (OFD + HA + PRP) 2005	Japan	35	35	Cafaclor 750 mg q.d. for 5 days	3.4 ±1.7	4.7 ± 1.6	3.5 ± 1.5
Yamamiya et al. (OFD + HA + PRP) 2008	Japan	15	15	Cafaclor 750 mg q.d. for 5 days	3.9 ± 1.6	4.8 ± 1.1	4.9 ± 1.2
Yassibag-Berkman et al. (OFD + bTCP + PRP) 2007	Turkey	-	10	Amoxicillin 875 + clavulanic 125 b.i.d. for 5 days	2.1 *	3.6 *	-
Paolantonio et al. (PPF + AB + l-PRF) 2020	Italy	22	22	Amoxicillin 875 mg + clavulanic 125 mg b.i.d. for 6 days	3.43 ± 0.74	4.21 ± 1.1	2.92 ± 0.71
**Other Combinations**							
Blumenthal et al. (OFD + AAA + CG) 1990	USA	-	12	Tetracycline 250 mg q.d. for 10 days	1.88 ± 0.2	2.61 ± 0.1	2.88 ± 0.2
Blumenthal et al. (OFD + AAA + CG + GTR-R) 1990	USA	-	15	Tetracycline 250 mg q.d. for 10 days	2.01 ± 0.1	2.73 ± 0.1	3.71 ± 0.1
Döri et al. (OFD + bTCP + GTR-NR + PRP) 2008a	Hungary	14	14	Amoxicillin 500 mg t.i.d. for 7 days	4.1 ± 0.7	5.8 ± 0.6	-
Christgau et al. (OFD + bTCP + GTR-R + PRP) 2006	Germany	25	25	Doxycycline 100 mg q.d. for 19 days	5 ± 1.5	6.3 ± 1.2	-
Yassibag-Berkman et al. (OFD + bTCP + PRP + GTR-R) 2007	Turkey	-	10	Amoxicillin 875 + clavulanic 125 b.i.d. for 5 days	2.4 *	4 *	-
Döri et al. (OFD + DBBM + EMD + PRP) 2008b	Hungary	13	13	Amoxicillin 500 mg t.i.d. for 7 days	4.8 ± 1.3	5.8 ± 1.8	-
Döri et al. (OFD + DBBM + GTR-NR + PRP) 2007a	Hungary	15	15	Amoxicillin 500 mg t.i.d. for 7 days	4.5 ± 1.1	5.5 ± 1.3	-
Döri et al. (OFD + DBBM + GTR-R + PRP) 2007b	Hungary	12	12	Amoxicillin 500 mg t.i.d. for 7 days	4.7 ± 1.1	5.5 ± 1.2	-
Minabe et al. (OFD + EMD + GTR-R) 2002	Japan	-	23	Cefaclor 750 mg q.d. for 4 days	3 ± 1.3	4.3 ± 1.6	-
Sipos et al. (OFD + EMD + GTR-R) 2005	Netherlands	11	12	No antibiotics	1.65 ± 1.29	3.02 ± 1.55	1.58 ± 1.92
Cetinkaya et al. (OFD + PP + GTR-R) 2014	Turkey	11	11	No antibiotics	2.36 ± 0.92	2.91 ± 0.94	3.45 ± 1.81
Aoki et al. (PPF + DBBM + rhFGF-2) 2020	Japan	20	20	Amoxicillin 750 mg or cefdinir 300 mg q.d. for 4 days	3.11 ± 1.46	3.58 ± 1.53	-
Ferrarotti et al. (PPF + DPSCs) 2018	Italy	15	15	Amoxicillin 875 + clavulanic 125, 1 g q.d. for 6 days	4.5 ± 1.9	4.9 ± 1.4	3.9 ± 1.5
Aoki et al. (PPF + rhFGF-2) 2020	Japan	18	18	Amoxicillin 750 mg or cefdinir 300 mg q.d. for 4 days	3.35 ± 1.28	3.58 ± 1.21	-
Santana et al. (PPF + rhFGF-2/HyAc) 2015	Brazil	30	30	Doxycycline 200 mg before surgery and 100 mg q.d. for 19 days	4.8 ± 0.2	5.5 ± 1.4	-

*: The standard deviation was not reported. §: The authors reported baseline and follow-up data but not the difference. AAA: autolyzed antigen-extracted allogenic freeze-dried bone; AB: autogenous bone; BG: bioactive glass; bTCP: Tricalcium Phosphate; CG: microfibrillar collagen gel; CPC: calcium phosphate bone cement; CS: calcium sulfate; CSM: calcium sulphate membrane; DBBM: demineralized bovine bone matrix; DFDBA: demineralized freeze-dried bone allograft; DPBM: demineralized porcine bone matrix; DPSCs: dental pulp stem cells; EMD: enamel matrix derivative; GTR-NR: guided tissue regeneration using a non-resorbable membrane; GTR-R: GTR using a resorbable membrane; HA: hydroxyapatite; HA/P-15: hydroxyapatite matrix/cell-binding peptide; HCPC: human cultured periosteum used in sheet as a membrane; HyAc: hyaluronic acid; l-PRF: leukocyte platelet-rich fibrin; OFD: open flap debridement; PDGF: platelet-derived growth factor; PLA/PGA polylactide/polyglicolide copolymer as biomaterial; PP: platelet pellet; PPF: papilla preservation flap; PRF: platelet-rich fibrin; rhFGF-2: recombinant human fibroblast growth factor.

**Table 2:** Characteristics of the treatment arms from included studies treating furcation defects.

Author/Treatment	Country	N° Patient	N° Defect	Antibiotic Regimen	V-CAL Gain	H-CAL Gain	PPD Reduction	H-BL Gain
**GTR−R**								
Blumenthal et al. (OFD + GTR-R) 1993	USA	12	12	Amoxicillin 500 mg t.i.d for 7 days	1.83 ± 1.47	2.5 ± 0.8	3.08 ± 1.68	2.5 ± 0.7
Bouchard et al. (OFD + GTR-R) 1993	France	12	12	Doxycycline 100 mg q.d. for 14 days	1.2 ± 1.8	1.5 ± 1.5	1.9 ± 2	1.5 ± 1.1
Maragos et al. (OFD + GTR-R) 2002	USA	-	11	Doxycycline 100 mg q.d. for 10 days	1.4 ± 0.4	-	-	0.9 ± 0.12
Pruthi et al. (OFD + GTR-R) 2002	Canada	17	17	Doxycycline 200 mg day of surgery and 100 mg q.d for 13 days	1 ± 1.22	-	1.47 ± 1.01	0.41 ± 0.71
Wang et al. (OFD + GTR-R) 1994	USA	12	12	Doxycycline 100 mg q.d. for 14 days	1.67 ± 0.76	-	2.8 ± 1.4	-
de Leonardis et al. (OFD + GTR-R) 1999	Italy/USA	12	12	Amoxicillin 875 mg + clavulanic 125 mg b.i.d. for 7 days	2 ± 1.3	-	2.4 ± 0.95	-
Garrett et al. (OFD + GTR-R) 1997	USA	66	66	Doxycycline 100 mg b.i.d. the day of surgery and q.d. for 13 days	2 ± 0.2	2.1 ± 0.4	2.3 ± 0.45	-
Bouchard et al. (OFD + GTR-R) 1997	France	15	15	Amoxicillin + clavulanic 1.5 g q.d. for 14 days	1.5 ± 1.9	2.5 ± 1.6	2.1 ± 1.6	-
Hugoson et al. (OFD + GTR-R) 1995	Sweden	38	38	No Antibiotics	0.4 ± 1.5	1.4 ± 2.2	2.2 ± 1.4	-
Karapataki et al. (OFD + GTR-R) 1999	Sweden	11	11	No antibiotics	1.1 ± 1.2	2.3 ± 2.23	-	-
Jepsen et al. (OFD + GTR-R) 2004	Germany	45	45	No Antibiotics	-	-	-	1.9 ± 1.4
**GTR-NR**								
Blumenthal et al. (OFD + GTR-NR) 1993	USA	12	12	Amoxicillin 500 mg t.i.d for 7 days	1.08 ± 0.79	1.83 ± 1.03	2.67 ± 1.15	1.7 ± 0.5
Bouchard et al. (OFD + GTR-NR) 1993	France	12	12	Doxycycline 100 mg q.d. for 14 days	1.3 ± 1.6	2.8 ± 1.3	2.2 ± 1.2	2.2 ± 1.4
Pruthi et al. (OFD + GTR-NR) 2002	Canada	17	17	Doxycycline 200 mg day of surgery and 100 mg q.d for 13 days	0.47 ± 1.81	-	1.12 ± 1.36	0.41 ± 0.62
Garrett et al. (OFD + GTR-NR) 1997	USA	64	64	Doxycycline 100 mg b.i.d. the day of surgery and q.d. for 13 days	1.6 ± 0.2	2.1 ± 0.4	2.1 ± 0.45	-
Bouchard et al. (OFD + GTR-NR) 1997	France	15	15	Amoxicillin + clavulanic 1.5 g q.d. for 14 days	1.2 ± 1.2	2.7 ± 1.2	1.8 ± 1.3	-
Hugoson et al. (OFD + GTR-NR) 1995	Sweden	38	38	No Antibiotics	0.8 ± 1.4	2.2 ± 2	2 ± 1	-
Karapataki et al. (OFD + GTR-NR) 1999	Sweden	11	11	No antibiotics	0.5 ± 1.2	0.5 ± 2.5	-	-
Leite et al. (OFD + GTR-NR) 2013 *	Brazil	12	12	Amoxicillin + clavulanic 500 mg t.i.d for 10 days starting the day before surgery	0.02 ± 1.32	-	0.43 ± 0.84	1.36 ± 1.57
Leite et al. (OFD + GTR-NR) 2013	Brazil	12	12	Amoxicillin + clavulanic 500 mg t.i.d for 10 days starting the day before surgery	0.24 ± 1.15	-	0.37 ± 1.03	1.18 ± 1.08
Villaça et al. (OFD + GTR-NR) 2004	Brazil	10	10	Amoxicillin + clavulanic 500 mg t.i.d for 10 days starting the day before surgery	§	-	§	§
Villaça et al. (OFD + GTR-NR) 2004 †	Brazil	10	10	Amoxicillin + clavulanic 500 mg t.i.d for 10 days starting the day before surgery	§	-	§	§
**Combinations**								
Maragos et al. (OFD + GTR-R + DFDBA) 2002	USA	-	14	Doxycycline 100 mg q.d. for 10 days	2.6 ± 0.3	-	-	2.2 ± 0.15
Jaiswal et al. (OFD + GTR-R + DFDBA) 2013	India	10	10	Amoxicillin 500 mg t.i.d for 7 days	0.85 ± 0.31	-	0.8 ± 0.72	-
de Leonardis et al. (OFD + GTR-R + DFDBA) 1999	Italy	12	12	Amoxicillin 875 mg + clavulanic 125 mg b.i.d. for 7 days	2.3 ± 0.85	-	2.8 ± 0.9	-
Garrett et al. (CAF + GTR-R + DFDBA) 1990	USA	13	15	Penicillin or erythromycin 250 mg q.i.d. for 14 days	-	-	2.2 ± 0.3	2 ± 2.1
Garrett et al. (OFD + DFDBA) 1990	USA	12	16	Penicillin or erythromycin 250 mg q.i.d. for 14 days	-	-	2.6 ± 1.13	2.6 ± 1.1
Gantes et al. (OFD + DFDBA) 1991	USA	13	13	Tetracycline 250 mg q.i.d for 14 days	∫	-	∫	-
Jaiswal et al. (OFD + GTR-R + DFDBA + EMD) 2013	India	10	10	Amoxicillin 500 mg t.i.d for 7 days	2.12 ± 1.07	-	1.74 ± 1	-
de Santana et al. (CAF + GTR-R + HA) 1999	USA	15	15	Doxycycline 200 mg day of surgery and 100 mg q.d for 19 days	1.57 ± 1.32	2.13 ± 1.52	2.43 ± 1.36	-
Santana et al. (CAF + GTR-NR + HA) 2009	Brazil	30	30	Doxycycline 200 mg day of surgery and 100 mg q.d for 19 days	3.05 ± 0.6	-	3.56 ± 0.6	-
Jepsen et al. (OFD + EMD) 2004	Germany	45	45	No Antibiotics	-	-	-	2.6 ± 1.8
Queiroz et al. (OFD + EMD) 2016	Brazil	13	13	No Antibiotics	2.08 ± 1.61	2.77 ± 0.93	2.54 ± 0.78	-
Queiroz et al. (OFD + bTCP/HA) 2016	Brazil	14	14	No Antibiotics	2.29 ± 1.27	2.64 ± 0.93	2.36 ± 1.01	-
Queiroz et al. (OFD + bTCP/HA + EMD) 2016	Brazil	14	14	No Antibiotics	2.14 ± 1.29	2.93 ± 0.83	2.43 ± 1.02	-

*: e-PTFE membrane was removed after 2 weeks. †: a modified e-PTFE membrane was used in this group. §: For this study were reported baseline and follow-up data but not the differences. ∫: At 12 months author reported only data on pocket closure. bTCP: Tricalcium Phosphate; CAF: Coronally advanced flap; DFDBA: demineralized freeze-dried bone allograft; EMD: enamel matrix derivative; GTR-NR: guided tissue regeneration using a non-resorbable membrane; GTR-R: GTR using a resorbable membrane; HA: hydroxyapatite; OFD: open flap debridement.

**Table 3:** Summary of meta-regressions for studies on ID.

Treatment	Number of Arms	Outcome	Difference	CI	*p*−Value
Overall/any treatment	AB− (32 arms)	PPD	−0.91 mm	−1.3 to −0.51	<0.001
	AB+ (82 arms)				
	AB− (32 arms)	CAL	−0.92 mm	−1.32 to −0.52	<0.001
	AB+ (82 arms)				
	AB− (15 arms)	BG	−1.1 mm	−1.63 to −0.53	<0.001
	AB+ (39 arms)				
EMD	AB− (13 arms)	PPD	−0.98 mm	−1.65 to −0.32	0.004
	AB+ (15 arms)				
	AB− (13 arms)	CAL	−0.98 mm	−1.77 to −0.19	0.015
	AB+ (15 arms)				
BRG + GTR-R	AB− (10 arms)	PPD	−0.22 mm	−0.94 to 0.5	0.553
	AB+ (23 arms)				
	AB− (10 arms)	CAL	−0.15 mm	−0.91 to 0.6	0.688
	AB+ (23 arms)				
Type of AB	Tetracycline (17 arms)	PPD	−0.25 mm	−0.79 to 0.29	0.360
	Penicillin (55 arms)				
	Tetracycline (17 arms)	CAL	−0.24 mm	−0.73 to 0.25	0.338
	Penicillin (55 arms)				

AB−: no antibiotic; AB+: antibiotic; PPD: probing pocket depth; CAL: clinical attachment level; BG: bone gain; EMD: enamel matrix derivative; BRG: bone replacement graft; GTR-R: guided tissue regeneration by mean resorbable membrane.

## Supplementary Materials

The following are available online at https://www.mdpi.com/article/10.3390/antibiotics11010008/s1, Material S1: PRISMA checklist; Material S2: Search strategy and study selection; Material S3: Full list of included papers; Material S4: Flow Chart diagram (modified from PRISMA); Material S5: Risk of bias; Material S6: Forest plots of single-arm meta-analyses and meta-regression models using AB as factor in intrabony defects; Material S7: Meta-regression using AB as factor in intrabony defects for the EMD subgroup; Material S8: Meta-regression using AB as factor in intrabony defects for the Graft +/− GTR-R subgroup; Material S9: Meta-regression using AB type as factor in intrabony defects; Material S10: Meta-regression using AB type as factor in furcation defects.
