# Supplementary material for: Adjunctive Effect of Systemic Antibiotics in Regenerative/Reconstructive Periodontal Surgery—A Systematic Review with Meta-Analysis
Source: Antibiotics (Basel). 2021 Dec 22;11(1):8. doi: 10.3390/antibiotics11010008 (PMC8772828; doi:10.3390/antibiotics11010008)
Supplement: Supplementary file 1 [file antibiotics-11-00008-s001.zip › antibiotics-1465072-supplementary.pdf]

Supplementary Material S1: PRISMA checklist.

Section	#	Checklist item	Reported on page
**TITLE**	1	Identify the report as systematic review, meta-analysis or both	1
**ABSTRACT**	2	Structured summary	2
**INRODUCTION**	3	Rationale	3
	4	Objectives	3
**METHODS**	5	Protocol and registration	3,4
	6	Eligibility criteria	4
	7	Information sources	4
	8	Search	4
	9	Study selection	4, 5
	10	Data collection process	5
	11	Data items	5
	12	Risk of bias in individual studies	5
	13	Summary measures	5
	14	Synthesis of results	5
	15	Risk of bias across studies	5
	16	Additional analyses	n/a
**RESULTS**	17	Study selection	6
	18	Study characteristics	6, Tables
	19	Risk of bias within studies	6, Suppl mat 5
	20	Results of individual studies	Tables
	21	Synthesis of results	6,7,8,9
	22	Risk of bias across studies	6
	23	Additional analysis	n/a
**DISCUSSION**	24	Summary of evidence	9,10,11,12

	25	Limitations	12
	26	Conclusions	12
**FUNDING**	27	Sources of funding	1

Supplementary Material S2: Search strategy and study selection.

The searches from two recent systematic reviews (SRs) (Nibali et al. 2020, Jepsen et al. 2020) were used as starting point for this SR. All the studies enclosed in the two SRs were reconsidered according to the PICO criteria for this review.

Intra-Bony Defect

In the review of Nibali et al. 2020, 88 manuscript reporting data for 79 RCTs were included. Eight manuscript reported data on long term follow-up from included RCTs (*Stravopolous 2010; Sculean 2007b; Hoffmann 2016; Sculean 2007a; Moder 2012; Döri 2013a; Döri 2013b; Mengel 2006*). These manuscripts were excluded from this review. The study of Nevins et al. 2013 was excluded as the data were reported on graphs and was not possible to retrieve the data expressed as mean and SD. The manuscript of Tonetti et al. 2004b reported data on post-operative healing and patient discomfort and was excluded also. (For the references see Nibali et al. 2020)

Thus, a total of 78 RCTs from those included in Nibali et al. 2020 were included in this SR.

Then, the list of excluded full-text from Nibali et al. 2020 was checked for additional title. Two articles were added from those excluded (*Abu-Ta'a 2016; Eickholz 2014*).

Finally, the electronic and hand searches used in Nibali et al. 2020 were updated from 31/01/2019 to 20/11/2020.

From the new research update 10 manuscript were selected to be evaluated full text according to the eligibility criteria for this SR:

- Four were excluded (*Corbella 2019* – Not RCT; *Kothiwale 2019* – less than 10 defects per group; *Xu 2019* – Unclear defect definition; *Sànchez 2020* Not RCT)
- Six were included in this systematic review (*Aslan 2020; Lee 2019; Mazzonetto 2020; Paolantonio 2020; Pietruska 2020; Aoki 2020*)

At the end of this process a total of 86 RCTs treating IDs with a regenerative approach were included in this SR.

Furcation Involvement

The 19 RCTs included in Jepsen et al. 2020, were all included in this SR.

The list of excluded full-text from Jepsen et al. 2020 was check but no additional titles were find.

The electronic and hand searches used in Jepsen et al. 2020 were updated from 01/12/2018 to 20/11/2020.

From the new research update 4 manuscript were selected to be evaluated full text according to the eligibility criteria for this SR. However, no manuscripts meet the inclusion criteria for this SR. (*Bevilacqua 2020*; less than 10 patients for group; *Djurkin 2019*, *Courval 2020* and *Shah 2019* less than 12 months follow-up)

At the end of this process a total of 19 RCTs treating FI with a regenerative approach were included in this SR.

1. Bevilacqua, L.; Fonzar, A.; Olivier, S.; De Biasi, M.; Visintin, M.; Angerame, D.; Maglione, M. Out-come of different surgical approaches in the treatment of class II furcation defects in mandibular molars: A randomized clinical trial. *Int. J. Periodontics Restor. Dent.* **2020**, *40*, 693–701.

2. Corbella, S.; Alberti, A.; Calciolari, E.; Taschieri, S.; Francetti, L. Enamel matrix derivative for the treatment of partially contained intrabony defects: 12-month results. Aust. Dent. J. 2019, 64, 27–34. https://doi.org/10.1111/adj.12654.

3. Courval, A.; Harmouche, L.; Mathieu, A.; Petit, C.; Huck, O.; Séverac, F.; Davideau, J.-L. Impact of Molar Furcations on Photodynamic Therapy Outcomes: A 6-Month Split-Mouth Randomized Clinical Trial. Int. J. Environ. Res. Public Health 2020, 17, 4162. https://doi.org/10.3390/ijerph17114162.

4. Djurkin, A.; Toma, S.; Brecx, M.C.; Lasserre, J.F. Treatment of mandibular Class II furcations using bovine-derived bone xenograft with or without a collagen membrane: A randomized controlled trial. Quintessence Int. 2019, 50, 652–660.

5. Kothiwale, S.; Bhimani, R.; Kaderi, M.; Ajbani, J. Comparative study of DFDBA and FDBA block grafts in combination with chorion membrane for the treatment of periodontal intra-bony defects at 12 months post surgery. Cell Tissue Bank. 2019, 1–9. https://doi.org/10.1007/s10561-018-09744-5.

6. Sànchez, N.; Fierravanti, L.; Núñez, J.; Vignoletti, F.; Gonzàlez-Zamora, M.; Santamaria, S.; Sanz, M. Periodontal regeneration using a xenogeneic bone substitute seeded with autologous periodon-tal ligament-derived mesenchymal stem cells: A 12-month quasi-randomized controlled pilot clinical trial. J. Clin. Periodontol. 2020, 47, 1391–1402.

7. Shah, K.K.; Kolte, R.A.; Kolte, A. Evaluation of Demineralized Freeze-Dried Bone Allograft in Combination with Chorion Membrane in the Treatment of Grade II Furcation Defects: A Randomized Controlled Trial. Int. J. Periodontics Restor. Dent. 2019, 39, 659–667. https://doi.org/10.11607/prd.4267.

8. Xu, Y.; Qiu, J.; Sun, Q.; Yan, S.; Wang, W.; Yang, P.; Song, A. One-Year Results Evaluating the Effects of Concentrated Growth Factors on the Healing of Intrabony Defects Treated with or without Bone Sub-stitute in Chronic Periodontitis. Med. Sci. Monit. 2019, 25, 4384–4389.

Supplementary Material S3: full list of included papers.

Intrabony Defect Studies

9. Abu-Ta'A, M. Adjunctive Systemic Antimicrobial Therapy vs Asepsis in Conjunction with Guided Tissue Regeneration: A Randomized, Controlled Clinical Trial. J. Contemp. Dent. Pr. 2016, 17, 3–6. https://doi.org/10.5005/jp-journals-10024-1794.

10. Agarwal, A.; Gupta, N.D. Platelet-rich plasma combined with decalcified freeze-dried bone allograft for the treatment of noncontained human intrabony periodontal defects: A randomized controlled split-mouth study. Int. J. Periodontics Restor. Dent. 2014, 34, 705–711. https://doi.org/10.11607/prd.1766.

11. Agarwal, A.; Gupta, N.D.; Jain, A. Platelet rich fibrin combined with decalcified freeze-dried bone allograft for the treatment of human intrabony periodontal defects: A randomized split mouth clinical trail. Acta Odontol. Scand. 2016, 74, 36–43. https://doi.org/10.3109/00016357.2015.1035672.

12. Al Machot, E.; Hoffmann, T.; Lorenz, K.; Khalili, I.; Noack, B. Clinical Outcomes after Treatment of Periodontal Intrabony Defects with Nanocrystalline Hydroxyapatite (Ostim) or Enamel Matrix Derivatives (Emdogain): A Randomized Controlled Clinical Trial. BioMed Res. Int. 2014, 2014, 1–9. https://doi.org/10.1155/2014/786353.

13. Aoki, H.; Bizenjima, T.; Seshima, F.; Sato, M.; Irokawa, D.; Yoshikawa, K.; Yoshida, W.; Imamura, K.; Matsugami, D.; Kitamura, Y.; et al. Periodontal surgery using rhFGF-2 with deproteinized bovine bone mineral or rhFGF-2 alone: 2-year follow-up of a randomized controlled trial. J. Clin. Periodontol. 2021, 48, 92–100. https://doi.org/10.1111/jcpe.13385.

14. Aslan, S.; Buduneli, N.; Cortellini, P. Clinical outcomes of the entire papilla preservation technique with and without biomaterials in the treatment of isolated intrabony defects: A randomized controlled clinical trial. J. Clin. Periodontol. 2020, 47, 470–478. https://doi.org/10.1111/jcpe.13255.

15. Aspriello, S.D.; Ferrante, L.; Rubini, C.; Piemontese, M. Comparative study of DFDBA in combination with enamel matrix derivative versus DFDBA alone for treatment of periodontal intrabony defects at 12 months post-surgery. Clin. Oral. Investig. 2010, 15, 225–232. https://doi.org/10.1007/s00784-009-0369-y.

16. Blumenthal, N.; Steinberg, J. The Use of Collagen Membrane Barriers in Conjunction With Combined Demineralized Bone-Collagen Gel Implants in Human Infrabony Defects. J. Periodontol. 1990, 61, 319–327. https://doi.org/10.1902/jop.1990.61.6.319.

17. Bokan, I.; Bill, J.S.; Schlagenhauf, U. Primary flap closure combined with Emdogain® alone or Emdogain® and Cerasorb® in the treatment of intra-bony defects. J. Clin. Periodontol. 2006, 33, 885–893. https://doi.org/10.1111/j.1600-051x.2006.01010.x.

18. Cetinkaya, B.O.; Keles, G.C.; Pamuk, F.; Balli, U.; Keles, Z.P. Long-term clinical results on the use of platelet concentrate in the treatment of intrabony periodontal defects. Acta Odontol. Scand. 2013, 72, 92–98. https://doi.org/10.3109/00016357.2013.775668.

19. Christgau, M.; Moder, D.; Wagner, J.; Gläßl, M.; Hiller, K.-A.; Wenzel, A.; Schmalz, G. Influence of autologous platelet concentrate on healing in intra-bony defects following guided tissue regeneration therapy: A randomized prospective clinical split-mouth study. J. Clin. Periodontol. 2006, 33, 908–921. https://doi.org/10.1111/j.1600-051x.2006.00999.x.

20. Christgau, M.; Schmalz, G.; Wenzel, A.; Hiller, K.A. Periodontal regeneration of intrabony defects with re-sorbable and non-resorbable membranes: 30-month results. J. Clin. Periodontol. 1997, 24, 17–27.

21. Cortellini, P.; Tonetti, M.S.; Lang, N.P.; Suvan, J.E.; Zucchelli, G.; Vangsted, T.; Silvestri, M.; Rossi, R.; McClain, P.; Fonzar, A.; et al. The Simplified Papilla Preservation Flap in the Regenerative Treatment of Deep Intrabony Defects: Clinical Outcomes and Postoperative Morbidity. J. Periodontol. 2001, 72, 1702–1712. https://doi.org/10.1902/jop.2001.72.12.1702.

22. Crea, A.; Dassatti, L.; Hoffmann, O.; Zafiropoulos, G.-G.; Deli, G. Treatment of Intrabony Defects Using Guided Tissue Regeneration or Enamel Matrix Derivative: A 3-Year Prospective Randomized Clinical Study. J. Periodontol. 2008, 79, 2281–2289. https://doi.org/10.1902/jop.2008.080135.

23. De Leonardis, D.; Paolantonio, M. Enamel Matrix Derivative, Alone or Associated With a Synthetic Bone Substitute, in the Treatment of 1- to 2-Wall Periodontal Defects. J. Periodontol. 2013, 84, 444–455. https://doi.org/10.1902/jop.2012.110656.

24. Santana, R.; De Santana, C.M.M. Human intrabony defect regeneration with rhFGF-2 and hyaluronic acid—A randomized controlled clinical trial. J. Clin. Periodontol. 2015, 42, 658–665. https://doi.org/10.1111/jcpe.12406.

25. Döri, F.; Arweiler, N.; Gera, I.; Sculean, A. Clinical Evaluation of an Enamel Matrix Protein Derivative Combined With Either a Natural Bone Mineral or β-Tricalcium Phosphate. J. Periodontol. 2005, 76, 2236–2243. https://doi.org/10.1902/jop.2005.76.12.2236.

26. Döri, F.; Húszár, T.; Nikolidakis, D.; Arweiler, N.B.; Gera, I.; Sculean, A. Effect of platelet-rich plasma on the healing of intra-bony defects treated with a natural bone mineral and a collagen membrane. J. Clin. Periodontol. 2007, 34, 254–261. https://doi.org/10.1111/j.1600-051x.2006.01044.x.

27. Döri, F.; Húszár, T.; Nikolidakis, D.; Arweiler, N.B.; Gera, I.; Sculean, A. Effect of Platelet-Rich Plasma on the Healing of Intrabony Defects Treated With an Anorganic Bovine Bone Mineral and Expanded Polytetrafluoroethylene Membranes. J. Periodontol. 2007, 78, 983–990. https://doi.org/10.1902/jop.2007.060349.

28. Döri, F.; Húszár, T.; Nikolidakis, D.; Tihanyi, D.; Horváth, A.; Arweiler, N.B.; Gera, I.; Sculean, A. Effect of Platelet-Rich Plasma on the Healing of Intrabony Defects Treated With Beta Tricalcium Phosphate and Expanded Polytetrafluoroethylene Membranes. J. Periodontol. 2008, 79, 660–669. https://doi.org/10.1902/jop.2008.070473.

29. Döri, F.; Kovács, V.; Arweiler, N.B.; Húszár, T.; Gera, I.; Nikolidakis, D.; Sculean, A. Effect of Platelet-Rich Plasma on the Healing of Intrabony Defects Treated With an Anorganic Bovine Bone Mineral: A Pilot Study. J. Periodontol. 2009, 80, 1599–1605. https://doi.org/10.1902/jop.2009.090058.

30. Döri, F.; Nikolidakis, D.; Húszár, T.; Arweiler, N.B.; Gera, I.; Sculean, A. Effect of platelet-rich plasma on the healing of intrabony defects treated with an enamel matrix protein derivative and a natural bone mineral. J. Clin. Periodontol. 2007, 35, 44–50. https://doi.org/10.1111/j.1600-051x.2007.01161.x.

31. Eickholz, P.; Röllke, L.; Schacher, B.; Wohlfeil, M.; Dannewitz, B.; Kaltschmitt, J.; Krieger, J.K.; Krigar, D.M.; Reitmeir, P.; Kim, T.-S. Enamel Matrix Derivative in Propylene Glycol Alginate for Treatment of Infrabony Defects With or Without Systemic Doxycycline: 12- and 24-Month Results. J. Periodontol. 2014, 85, 669–675. https://doi.org/10.1902/jop.2013.130290.

32. Ferrarotti, F.; Romano, F.; Gamba, M.N.; Quirico, A.; Giraudi, M.; Audagna, M.; Aimetti, M. Human intrabony defect regeneration with micrografts containing dental pulp stem cells: A randomized controlled clinical trial. J. Clin. Periodontol. 2018, 45, 841–850. https://doi.org/10.1111/jcpe.12931.

33. Fickl, S.; Thalmair, T.; Kebschull, M.; Böhm, S.; Wachtel, H. Microsurgical access flap in conjunction with enamel matrix derivative for the treatment of intra-bony defects: A controlled clinical trial. J. Clin. Periodontol. 2009, 36, 784–790. https://doi.org/10.1111/j.1600-051x.2009.01451.x.

34. Francetti, L.; Del Fabbro, M.; Basso, M.; Testori, T.; Weinstein, R. Enamel matrix proteins in the treatment of intra-bony defects. A prospective 24-month clinical trial. J. Clin. Periodontol. 2003, 31, 52–59. https://doi.org/10.1111/j.0303-6979.2004.00437.x.

35. Francetti, L.; Trombelli, L.; Lombardo, G.; Guida, L.; Cafiero, C.; Roccuzzo, M.; Carusi, G.; Del Fabbro, M. Evaluation of efficacy of enamel matrix derivative in the treatment of intrabony defects: A 24-month multicenter study. Int. J. Periodontics Restor. Dent. 2005, 25, 461–73.

36. Ghezzi, C.; Ferrantino, L.; Bernardini, L.; Lencioni, M.; Masiero, S. Minimally Invasive Surgical Technique in Periodontal Regeneration: A Randomized Controlled Clinical Trial Pilot Study. Int. J. Periodontics Restor. Dent. 2016, 36, 475–482. https://doi.org/10.11607/prd.2550.

37. Rathe, F.; Junker, R.; Chesnutt, B.M.; Jansen, J.A. The Effect of Enamel Matrix Derivative (Emdogain®) on Bone Formation: A Systematic Review. Tissue Eng. Part B Rev. 2009, 15, 215–224. https://doi.org/10.1089/ten.teb.2008.0065.

38. Guida, L.; Annunziata, M.; Belardo, S.; Farina, R.; Scabbia, A.; Trombelli, L. Effect of Autogenous Cortical Bone Particulate in Conjunction With Enamel Matrix Derivative in the Treatment of Periodontal Intraosseous Defects. J. Periodontol. 2007, 78, 231–238. https://doi.org/10.1902/jop.2007.060142.

39. Heijl, L.; Heden, G.; Svärdström, G.; Ostgren, A. Enamel matrix derivative (EMDOGAIN) in the treatment of intrabony periodontal defects. J. Clin. Periodontol. 1997, 24, 705–714.

40. Iorio-Siciliano, V.; Andreuccetti, G.; Blasi, A.; Matarasso, M.; Sculean, A.; Salvi, G.E. Clinical Outcomes Following Regenerative Therapy of Non-Contained Intrabony Defects Using a Deproteinized Bovine Bone Mineral Combined With Either Enamel Matrix Derivative or Collagen Membrane. J. Periodontol. 2014, 85, 1342–1350. https://doi.org/10.1902/jop.2014.130420.

41. Siciliano, V.I.; Andreuccetti, G.; Siciliano, A.I.; Blasi, A.; Sculean, A.; Salvi, G.E. Clinical Outcomes After Treatment of Non-Contained Intrabony Defects With Enamel Matrix Derivative or Guided Tissue Regeneration: A 12-Month Randomized Controlled Clinical Trial. J. Periodontol. 2011, 82, 62–71. https://doi.org/10.1902/jop.2010.100144.

42. Kasaj, A.; Röhrig, B.; Reichert, C.; Willershausen, B. Clinical evaluation of anorganic bovine-derived hydroxyapatite matrix/cell-binding peptide (P-15) in the treatment of human infrabony defects. Clin. Oral. Investig. 2008, 12, 241–247. https://doi.org/10.1007/s00784-008-0191-y.

43. Kim, C.-K.; Chai, J.-K.; Cho, K.-S.; Moon, I.-S.; Choi, S.-H.; Sottosanti, J.S.; Wikesjö, U.M. Periodontal Repair in Intrabony Defects Treated With a Calcium Sulfate Implant and Calcium Sulfate Barrier. J. Periodontol. 1998, 69, 1317–1324. https://doi.org/10.1902/jop.1998.69.12.1317.

44. Lee, J.; Kim, D.; Jeong, S. Adjunctive use of enamel matrix derivatives to porcine-derived xenograft for the treatment of one-wall intrabony defects: Two-year longitudinal results of a randomized controlled clinical trial. J. Periodontol. 2019, 91, 880–889. https://doi.org/10.1002/jper.19-0432.

45. Leknes, K.N.; Andersen, K.-M.; Bøe, O.E.; Skavland, R.J.; Albandar, J.M. Enamel Matrix Derivative Versus Bioactive Ceramic Filler in the Treatment of Intrabony Defects: 12-Month Results. J. Periodontol. 2009, 80, 219–227. https://doi.org/10.1902/jop.2009.080236.

46. Loos, B.G.; Louwerse, P.H.G.; Van Winkelhoff, A.J.; Burger, W.; Gilijamse, M.; Hart, A.A.M.; Van Der Velden, U. Use of barrier membranes and systemic antibiotics in the treatment of intraosseous defects. J. Clin. Periodontol. 2002, 29, 910–921. https://doi.org/10.1034/j.1600-051x.2002.291006.x.

47. Losada, M.; González, R.; Garcia, À.P.; Santos, A.; Nart, J.; Pujol, À. Treatment of Non-Contained Infrabony Defects With Enamel Matrix Derivative Alone or in Combination With Biphasic Calcium Phosphate Bone Graft: A 12-Month Randomized Controlled Clinical Trial. J. Periodontol. 2017, 88, 426–435. https://doi.org/10.1902/jop.2016.160459.

48. Mayfield, L.; Söderholm, G.; Hallström, H.; Kullendorff, B.; Edwardsson, S.; Bratthall, G.; Brägger, U.; Attström, R. Guided tissue regeneration for the treatment of intraosseous defects using a bioab-sorbable membrane. A controlled clinical study. J. Clin. Periodontol. 1998, 25, 585–595.

49. Mazzonetto, A.L.F.; Casarin, R.C.V.; Santamaria, M.P.; Andere, N.M.R.B.; Araújo, C.F.; da Silva, R.V.C.; Purisaca, J.E.V.; Sallum, E.A.; Sallum, A.W. Clinical, radiographic, and patient-centered outcomes after use of enamel matrix proteins for the treatment of intrabony defects in patients with aggressive periodontitis: A 12-month multicenter clinical trial. J. Periodontol. 2021, 92, 995–1006. https://doi.org/10.1002/jper.20-0493.

50. Mengel, R.; Soffner, M.; Flores-De-Jacoby, L. Bioabsorbable Membrane and Bioactive Glass in the Treatment of Intrabony Defects in Patients with Generalized Aggressive Periodontitis: Results of a 12-Month Clinical and Radiological Study. J. Periodontol. 2003, 74, 899–908. https://doi.org/10.1902/jop.2003.74.6.899.

51. Meyle, J.; Hoffmann, T.; Topoll, H.; Heinz, B.; Al-Machot, E.; Jervøe-Storm, P.-M.; Meiß, C.; Eickholz, P.; Jepsen, S. A multi-centre randomized controlled clinical trial on the treatment of intra-bony defects with enamel matrix derivatives/synthetic bone graft or enamel matrix derivatives alone: Results after 12 months. J. Clin. Periodontol. 2011, 38, 652–660. https://doi.org/10.1111/j.1600-051x.2011.01726.x.

52. Minabe, M.; Kodama, T.; Kogou, T.; Takeuchi, K.; Fushimi, H.; Sugiyama, T.; Mitarai, E. A comparative study of combined treatment with a collagen membrane and enamel matrix proteins for the regeneration of intraosseous defects. Int. J. Periodontics Restor. Dent. 2002, 22, 595–605.

53. Minenna, L.; Herrero, F.; Sanz, M.; Trombelli, L. Adjunctive effect of a polylactide/polyglycolide copolymer in the treatment of deep periodontal intra-osseous defects: A randomized clinical trial. J. Clin. Periodontol. 2005, 32, 456–461. https://doi.org/10.1111/j.1600-051x.2005.00696.x.

54. Moher, D.; Liberati, A.; Tetzlaff, J.; Altman, D.G. Preferred Reporting Items for Systematic Reviews and Meta-Analyses: The PRISMA Statement. J. Clin. Epidemiol. 2009, 62, 1006–1012. https://doi.org/10.1016/j.jclinepi.2009.06.005.

55. Mora, F.; Etienne, D.; Ouhayoun, J. Treatment of interproximal angular defects by guided tissue regeneration: 1 year follow-up. J. Oral. Rehabil. 1996, 23, 599–606. https://doi.org/10.1046/j.1365-2842.1996.d01-206.x.

56. Nygaard-Østby, P.; Bakke, V.; Nesdal, O.; Susin, C.; Wikesjö, U.M.E. Periodontal healing following reconstructive surgery: Effect of guided tissue regeneration using a bioresorbable barrier device when combined with autogenous bone grafting. A randomized-controlled trial 10-year follow-up. J. Clin. Periodontol. 2010, 37, 366–373. https://doi.org/10.1111/j.1600-051x.2010.01532.x.

57. Okuda, K.; Momose, M.; Miyazaki, A.; Murata, M.; Yokoyama, S.; Yonezawa, Y.; Wolff, L.F.; Yoshie, H. Enamel Matrix Derivative in the Treatment of Human Intrabony Osseous Defects. J. Periodontol. 2000, 71, 1821–1828. https://doi.org/10.1902/jop.2000.71.12.1821.

58. Okuda, K.; Tai, H.; Tanabe, K.; Suzuki, H.; Sato, T.; Kawase, T.; Saito, Y.; Wolff, L.F.; Yoshiex, H. Platelet-Rich Plasma Combined With a Porous Hydroxyapatite Graft for the Treatment of Intrabony Periodontal Defects in Humans: A Comparative Controlled Clinical Study. J. Periodontol. 2005, 76, 890–898. https://doi.org/10.1902/jop.2005.76.6.890.

59. Orsini, M.; Orsini, G.; Benlloch, D.; Aranda, J.J.; Sanz, M. Long-Term Clinical Results on the Use of Bone-Replacement Grafts in the Treatment of Intrabony Periodontal Defects. Comparison of the Use of Autogenous Bone Graft Plus Calcium Sulfate to Autogenous Bone Graft Covered With a Bioabsorbable Membrane. J. Periodontol. 2008, 79, 1630–1637. https://doi.org/10.1902/jop.2008.070282.

60. Paolantonio, M. Combined Periodontal Regenerative Technique in Human Intrabony Defects by Collagen Membranes and Anorganic Bovine Bone. A Controlled Clinical Study. J. Periodontol. 2002, 73, 158–166. https://doi.org/10.1902/jop.2002.73.2.158.

61. Paolantonio, M.; Perinetti, G.; Dolci, M.; Perfetti, G.; Tetè, S.; Sammartino, G.; Femminella, B.; Graziani, F. Surgical Treatment of Periodontal Intrabony Defects With Calcium Sulfate Implant and Barrier Versus Collagen Barrier or Open Flap Debridement Alone: A 12-Month Randomized Controlled Clinical Trial. J. Periodontol. 2008, 79, 1886–1893. https://doi.org/10.1902/jop.2008.080076.

62. Paolantonio, M.; Di Tullio, M.; Giraudi, M.; Romano, L.; Secondi, L.; Paolantonio, G.; Graziani, F.; Pilloni, A.; De Ninis, P.; Femminella, B. Periodontal regeneration by leukocyte and platelet-rich fibrin with autogenous bone graft versus enamel matrix derivative with autogenous bone graft in the treatment of periodontal intrabony defects: A randomized non-inferiority trial. J. Periodontol. 2020, 91, 1595–1608. https://doi.org/10.1002/jper.19-0533.

63. Patel, G.K.; Gaekwad, S.S.; Gujjari, S.K.; S.C., V.K. Platelet-Rich Fibrin in Regeneration of Intrabony Defects: A Randomized Controlled Trial. J. Periodontol. 2017, 88, 1192–1199. https://doi.org/10.1902/jop.2017.130710.

64. Piemontese, M.; Aspriello, S.D.; Rubini, C.; Ferrante, L.; Procaccini, M. Treatment of Periodontal Intrabony Defects With Demineralized Freeze-Dried Bone Allograft in Combination With Platelet-Rich Plasma: A Comparative Clinical Trial. J. Periodontol. 2008, 79, 802–810. https://doi.org/10.1902/jop.2008.070436.

65. Pietruska, M.; Pietruski, J.; Nagy, K.; Brecx, M.; Arweiler, N.B.; Sculean, A. Four-year results following treatment of intrabony periodontal defects with an enamel matrix derivative alone or combined with a biphasic calcium phosphate. Clin. Oral. Investig. 2011, 16, 1191–1197. https://doi.org/10.1007/s00784-011-0611-2.

66. Pietruska, M.; Skurska, A.; Pietruski, J.; Dolińska, E.; Arweiler, N.; Milewski, R.; Duraj, E.; Sculean, A. Clinical and radiographic evaluation of intrabony periodontal defect treatment by open flap debridement alone or in combination with nanocrystalline hydroxyapatite bone substitute. Ann. Anat. 2012, 194, 533–537. https://doi.org/10.1016/j.aanat.2012.03.009.

67. Pontoriero, R.; Wennström, J.; Lindhe, J. The use of barrier membranes and enamel matrix proteins in the treatment of angular bone defects. A prospective controlled clinical study. J. Clin. Perio. Dontol. 1999, 26, 833–840.

68. Qiao, J.; Duan, J.; Zhang, Y.; Chu, Y.; Sun, C. The effect of concentrated growth factors in the treatment of periodontal intrabony defects. Futur. Sci. OA 2016, 2, FS136. https://doi.org/10.4155/fsoa-2016-0019.

69. Rösing, C.K.; Aass, A.M.; Mavropoulos, A.; Gjermo, P. Clinical and Radiographic Effects of Enamel Matrix Derivative in the Treatment of Intrabony Periodontal Defects: A 12-Month Longitudinal Placebo-Controlled Clinical Trial in Adult Periodontitis Patients. J. Periodontol. 2005, 76, 129–133. https://doi.org/10.1902/jop.2005.76.1.129.

70. Sanz, M.; Tonetti, M.S.; Zabalegui, I.; Sicilia, A.; Blanco, J.; Rebelo, H.; Rasperini, G.; Merli, M.; Cortellini, P.; Suvan, J.E. Treatment of Intrabony Defects With Enamel Matrix Proteins or Barrier Membranes: Results From a Multicenter Practice-Based Clinical Trial. J. Periodontol. 2004, 75, 726–733. https://doi.org/10.1902/jop.2004.75.5.726.

71. Scabbia, A.; Trombelli, L. A comparative study on the use of a HA/collagen/chondroitin sulphate biomaterial (BiostiteR) and a bovine-derived HA xenograft (Bio-OssR) in the treatment of deep intra-osseous defects. J. Clin. Periodontol. 2004, 31, 348–355. https://doi.org/10.1111/j.1600-051x.2004.00483.x.

72. Sculean, A.; Barbé, G.; Chiantella, G.C.; Arweiler, N.B.; Berakdar, M.; Brecx, M. Clinical Evaluation of an Enamel Matrix Protein Derivative Combined With a Bioactive Glass for the Treatment of Intrabony Periodontal Defects in Humans. J. Periodontol. 2002, 73, 401–408. https://doi.org/10.1902/jop.2002.73.4.401.

73. Sculean, A.; Berakdar, M.; Chiantella, G.C.; Donos, N.; Arweiler, N.B.; Brecx, M. Healing of intrabony defects following treatment with a bovine-derived xenograft and collagen membrane. J. Clin. Periodontol. 2003, 30, 73–80. https://doi.org/10.1034/j.1600-051x.2003.10192.x.

74. Sculean, A.; Chiantella, G.C.; Windisch, P.; Arweiler, N.B.; Brecx, M.; Gera, I. Healing of intra-bony defects following treatment with a composite bovine-derived xenograft (Bio-Oss Collagen) in combination with a collagen membrane (Bio-Gide PERIO). J. Clin. Periodontol. 2005, 32, 720–724. https://doi.org/10.1111/j.1600-051x.2005.00758.x.

75. Sculean, A.; Chiantella, G.C.; Windisch, P.; Gera, I.; Reich, E. Clinical evaluation of an enamel matrix protein derivative (Emdogain) combined with a bovine-derived xenograft (Bio-Oss) for the treatment of intrabony periodontal defects in humans. Int. J. Periodontics Restor. Dent. 2002, 22, 259–67.

76. Sculean, A.; Nikolidakis, D.; Nikou, G.; Ivanovic, A.; Chapple, I.L.C.; Stavropoulos, A. Biomaterials for promoting periodontal regeneration in human intrabony defects: A systematic review. Periodontology 2000 2015, 68, 182–216. https://doi.org/10.1111/prd.12086.

77. Sculean, A.; Pietruska, M.; Schwarz, F.; Willershausen, B.; Arweiler, N.B.; Auschill, T.M. Healing of human intrabony defects following regenerative periodontal therapy with an enamel matrix protein derivative alone or combined with a bioactive glass. A controlled clinical study. J. Clin. Periodontol. 2005, 32, 111–117. https://doi.org/10.1111/j.1600-051x.2004.00635.x.

78. Shirakata, Y.; Setoguchi, T.; Machigashira, M.; Matsuyama, T.; Furuichi, Y.; Hasegawa, K.; Yoshimoto, T.; Izumi, Y. Comparison of Injectable Calcium Phosphate Bone Cement Grafting and Open Flap Debridement in Periodontal Intrabony Defects: A Randomized Clinical Trial. J. Periodontol. 2008, 79, 25–32. https://doi.org/10.1902/jop.2008.070141.

79. Silvestri, M.; Ricci, G.; Rasperini, G.; Sartori, S.; Cattaneo, V. Comparison of treatments of infrabony defects with enamel matrix derivative, guided tissue regeneration with a nonresorbable membrane and Widman modified flap. A pilot study. J. Clin. Periodontol. 2000, 27, 603–610.

80. Silvestri, M.; Sartori, S.; Rasperini, G.; Ricci, G.; Rota, C.; Cattaneo, V. Comparison of infrabony de-fects treated with enamel matrix derivative versus guided tissue regeneration with a nonresorbable membrane. J. Clin. Periodontol. 2003, 30, 386–393.

81. Sipos, P.M.; Loos, B.G.; Abbas, F.; Timmerman, M.F.; Van Der Velden, U. The combined use of enamel matrix proteins and a tetracycline-coated expanded polytetrafluoroethylene barrier membrane in the treatment of intra-osseous defects. J. Clin. Periodontol. 2005, 32, 765–772. https://doi.org/10.1111/j.1600-051x.2005.00754.x.

82. Slotte, C.; Asklöw, B.; Sultan, J.; Norderyd, O. A Randomized Study of Open-Flap Surgery of 32 Intrabony Defects With and Without Adjunct Bovine Bone Mineral Treatment. J. Periodontol. 2012, 83, 999–1007. https://doi.org/10.1902/jop.2011.110490.

83. Stavropoulos, A.; Karring, E.S.; Kostopoulos, L.; Karring, T. Deproteinized bovine bone and gentami-cin as an adjunct to GTR in the treatment of intrabony defects: A randomized controlled clinical study. J. Clin. Periodontol. 2003, 30, 486–495.

84. Thorat, M.; Baghele, O.; Rakhewar, P.S. Adjunctive Effect of Autologous Platelet-Rich Fibrin in the Treatment of Intrabony Defects in Localized Aggressive Periodontitis Patients: A Randomized Controlled Split-Mouth Clinical Trial. Int. J. Periodontics Restor. Dent. 2017, 37, e302–e309. https://doi.org/10.11607/prd.2972.

85. Tonetti, M.S.; Cortellini, P.; Lang, N.P.; Suvan, J.E.; Adriaens, P.; Dubravec, D.; Fonzar, A.; Fourmousis, I.; Rasperini, G.; Rossi, R.; et al. Clinical outcomes following treatment of human intrabony defects with GTR/bone replacement material or access flap alone. A multicenter randomized controlled clinical trial. J. Clin. Periodontol. 2004, 31, 770–776. https://doi.org/10.1111/j.1600-051x.2004.00562.x.

86. Tonetti, M.S.; Cortellini, P.; Suvan, J.E.; Adriaens, P.; Baldi, C.; Dubravec, D.; Fonzar, A.; Fourmousis, I.; Magnani, C.; Muller-Campanile, V.; et al. Generalizability of the Added Benefits of Guided Tissue Regeneration in the Treatment of Deep Intrabony Defects. Evaluation in a Multi-Center Randomized Controlled Clinical Trial. J. Periodontol. 1998, 69, 1183–1192. https://doi.org/10.1902/jop.1998.69.11.1183.

87. Tonetti, M.S.; Lang, N.P.; Cortellini, P.; Suvan, J.E.; Adriaens, P.; Dubravec, D.; Fonzar, A.; Fourmousis, I.; Mayfield, L.; Rossi, R.; et al. Enamel matrix proteins in the regenerative therapy of deep intrabony defects. J. Clin. Periodontol. 2002, 29, 317–325. https://doi.org/10.1034/j.1600-051x.2002.290407.x.

88. Trejo, P.; Weltman, R.; Caffesse, R. Treatment of Intraosseous Defects with Bioabsorbable Barriers Alone or in Combination With Decalcified Freeze-Dried Bone Allograft: A Randomized Clinical Trial. J. Periodontol. 2000, 71, 1852–1861. https://doi.org/10.1902/jop.2000.71.12.1852.

89. Wachtel, H.; Schenk, G.; Böhm, S.; Weng, D.; Zuhr, O.; Hürzeler, M.B. Microsurgical access flap and enamel matrix derivative for the treatment of periodontal intrabony defects: A controlled clinical study. J. Clin. Periodontol. 2003, 30, 496–504. https://doi.org/10.1034/j.1600-051x.2003.00013.x.

90. Yamamiya, K.; Okuda, K.; Kawase, T.; Hata, K.-I.; Wolff, L.F.; Yoshie, H. Tissue-Engineered Cultured Periosteum Used With Platelet-Rich Plasma and Hydroxyapatite in Treating Human Osseous Defects. J. Periodontol. 2008, 79, 811–818. https://doi.org/10.1902/jop.2008.070518.

91. Yassibag-Berkman, Z.; Tuncer, O.; Subasioglu, T.; Kantarci, A. Combined Use of Platelet-Rich Plasma and Bone Grafting With or Without Guided Tissue Regeneration in the Treatment of Anterior Interproximal Defects. J. Periodontol. 2007, 78, 801–809. https://doi.org/10.1902/jop.2007.060318.

92. Yen, C.-C.; Tu, Y.-K.; Chen, T.-H.; Lu, H.-K. Comparison of treatment effects of guided tissue regeneration on infrabony lesions between animal and human studies: A systematic review and meta-analysis. J. Periodontal Res. 2013, 49, 415–424. https://doi.org/10.1111/jre.12130.

93. Yilmaz, S.; Cakar, G.; Yildirim, B.; Sculean, A. Healing of two and three wall intrabony periodontal defects following treatment with an enamel matrix derivative combined with autogenous bone. J. Clin. Periodontol. 2010, 37, 544–550. https://doi.org/10.1111/j.1600-051x.2010.01567.x.

94. Zucchelli, G.; Amore, C.; Montebugnoli, L.; De Sanctis, M. Enamel Matrix Protines Bovine Porous Bone Mineral in the Treatment of Intrabony Defects: Comparative Controlled Clinical Trial. J. Periodontol. 2003, 74, 1725–1735. https://doi.org/10.1902/jop.2003.74.12.1725.

95. Zucchelli, G.; Bernardi, F.; Montebugnoli, L.; De Sanctis, M. Enamel Matrix Proteins and Guided Tissue Regeneration With Titanium-Reinforced Expanded PolytetrafluoroethyleneMembranes in the Treatment of Infrabony Defects: A Comparative Controlled Clinical Trial. J. Periodontol. 2002, 73, 3–12. https://doi.org/10.1902/jop.2002.73.1.3.

Furcation Studies

96. Zybutz, M.D.; Laurell, L.; Rapoport, D.A.; Persson, G.R. Treatment of intrabony defects with resorb-able materials, non-resorbable materials and flap debridement. J. Clin. Periodontol. 2000, 27, 169–178.

97. Blumenthal, N.M. A Clinical Comparison of Collagen Membranes With e-PTFE Membranes in the Treatment of Human Mandibular Buccal Class II Furcation Defects. J. Periodontol. 1993, 64, 925–933. https://doi.org/10.1902/jop.1993.64.10.925.

98. Bouchard, P.; Giovannoli, J.-L.; Mattout, C.; Davarpanah, M.; Etienne, D. Clinical evaluation of a bioabsorbable regenerative material in mandibular class II furcation therapy. J. Clin. Periodontol. 1997, 24, 511–518. https://doi.org/10.1111/j.1600-051x.1997.tb00220.x.

99. Bouchard, P.; Ouhayoun, J.P.; Nilvéus, R.E. Expanded polytet- rafluoroethylene membranes and connective tissue grafts support bone regeneration for closing mandibular Class II furcations. J. Periodontol. 1993, 64, 1193–1198. https://doi.org/10.1902/ jop.1993.64.12.1193.

100. De Leonardis, D.; Garg, A.; Pedrazzoli, V.; Pecora, G. Clinical Evaluation of the Treatment of Class II Furcation Involvements With Bioabsorbable Barriers Alone or Associated With Demineralized Freeze-Dried Bone Allografts. J. Periodontol. 1999, 70, 8–12. https://doi.org/10.1902/jop.1999.70.1.8.

101. de Santana, R.B.; Gusman, H.C.; Van Dyke, T.E. The response of human buccal maxillary fur-cation defects to combined regenerative techniques–two controlled clinical studies. J. Int. Acad. Periodontol. 1999, 1, 69–77.

102. Gantés, B.G.; Synowski, B.N.; Garrett, S.; Egelberg, J.H. Treatment of Periodontal Furcation Defects. Mandibular Class III Defects. J. Periodontol. 1991, 62, 361–365. https://doi.org/10.1902/jop.1991.62.6.361.

103. Garrett, S.; Martin, M.; Egelberg, J. Treatment of periodontal furcation defects Coronally positioned flaps versus dura mater membranes in class II defects. J. Clin. Periodontol. 1990, 17, 179–185. https://doi.org/10.1111/j.1600-051x.1990.tb01083.x.

104. Garrett, S.; Polson, A.M.; Stoller, N.H.; Drisko, C.L.; Caton, J.G.; Harrold, C.Q.; DeRouen, T.A. Comparison of a bioabsorbable GTR barrier to a non-absorbable barrier in treating human class II furca- tion defects. A multi-center parallel design randomized single-blind trial. J. Periodontol. 1997, 68, 667–675. https://doi.org/10.1902/ jop.1997.68.7.667.

105. Hugoson, A.; Ravald, N.; Fornell, J.; Johard, G.; Teiwik, A.; Gottlow, J. Treatment of Class II Furcation Involvements in Humans With Bioresorbable and Nonresorbable Guided Tissue Regeneration Barriers. A Randomized Multi-Center Study. J. Periodontol. 1995, 66, 624–634. https://doi.org/10.1902/jop.1995.66.7.624.

106. Jaiswal, R.; Deo, V. Evaluation of the effectiveness of enamel matrix derivative, bone grafts, and membrane in the treatment of mandibular Class II furcation defects. Int. J. Periodontics Restor. Dent. 2013, 33, e58–e64. https://doi.org/10.11607/prd.1428.

107. Jepsen, S.; Heinz, B.; Jepsen, K.; Arjomand, M.; Hoffmann, T.; Richter, S.; Reich, E.; Sculean, A.; Gonzales, J.R.; Bödeker, R.H.; et al. A Randomized Clinical Trial Comparing Enamel Matrix Derivative and Membrane Treatment of Buccal Class II Furcation Involvement in Mandibular Molars. Part I: Study Design and Results for Primary Outcomes. J. Periodontol. 2004, 75, 1150–1160. https://doi.org/10.1902/jop.2004.75.8.1150.

108. Karapataki, S.; Falk, H.; Hugoso, A.; Olsson, G.; Slotte, C. Treatment of class II furcation defects using resorbable and non-re- sorbable GTR barriers. Swed. Dent. J. 1999, 23, 173–183.

109. Leite, A.C.; De Oliveira, R.R.; Novaes, A.B., Jr.; O'Connell, P.A.; Grisi, M.F.M.; Taba, M., Jr.; Palioto, D.B.; Souza, S.L.S. Effect of Early Membrane Removal on the Treatment of Mandibular Class II Furcation Defects—A Controlled Clinical Trial with Re-entry after 12 Months. Braz. Dent. J. 2013, 24, 402–409. https://doi.org/10.1590/0103-6440201302243.

110. Maragos, P.; Bissada, N.F.; Wang, R.; Cole, B.P. Comparison of three methods using calcium sulfate as a graft/barrier material for the treatment of Class II mandibular molar furcation defects. Int. J. Periodontics Restor. Dent. 2002, 22, 493–501.

111. Pruthi, V.K.; Gelskey, S.C.; Mirbod, S.M. Furcation therapy with bioabsorbable collagen membrane: A clinical trial. J. Can. Dent. Assoc. 2002, 68, 610–615.

112. Queiroz, L.A.; Santamaria, M.P.; Casati, M.Z.; Ruiz, K.S.; Nociti, F.; Sallum, A.W.; Sallum, E.A. Enamel matrix protein derivative and/or synthetic bone substitute for the treatment of mandibular class II buccal furcation defects. A 12-month randomized clinical trial. Clin. Oral Investig. 2015, 20, 1597–1606. https://doi.org/10.1007/s00784-015-1642-x.

113. Santana, R.; De Mattos, C.M.L.; Van Dyke, T. Efficacy of Combined Regenerative Treatments in Human Mandibular Class II Furcation Defects. J. Periodontol. 2009, 80, 1756–1764. https://doi.org/10.1902/jop.2009.080605.

114. Villaça, J.H.; Rodrigues, D.C.; Novaes, A.B.; Taba, M.; Souza, S.L.; Grisi, M.F. Root trunk con-cavities as a risk factor for regenerative procedures of class II furcation lesions in humans. J. Periodontol. 2004, 75, 1493–1499. https://doi.org/10.1902/ jop.2004.75.11.1493.

115. Wang, H.-L.; O'Neal, R.B.; Thomas, C.L.; Shyr, Y.; MacNeil, R.L. Evaluation of an Absorbable Collagen Membrane in Treating Class II Furcation Defects. J. Periodontol. 1994, 65, 1029–1036. https://doi.org/10.1902/jop.1994.65.11.1029.

Supplementary Material S4: Flow Chart diagram (modified from PRISMA).

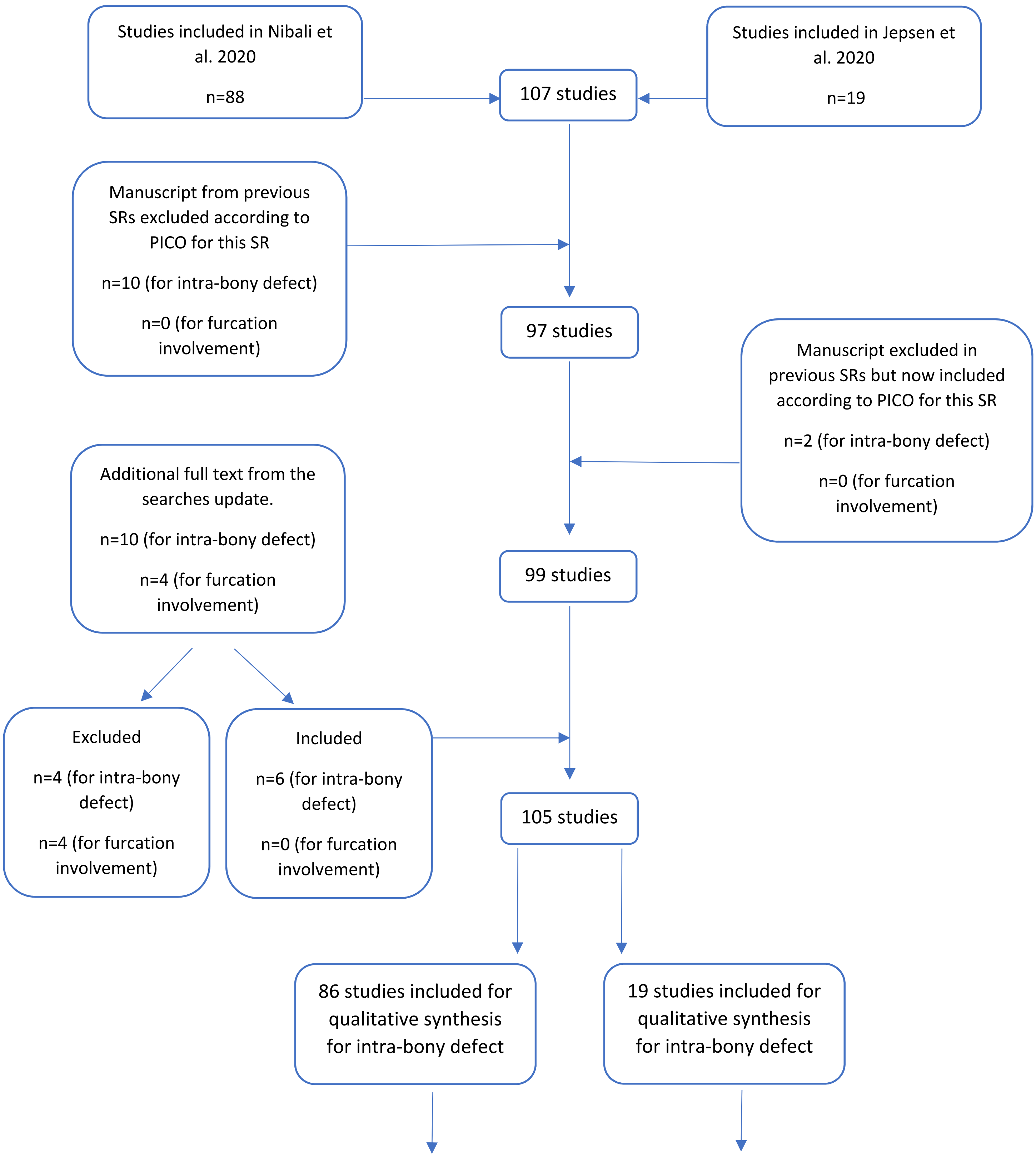

70 studies included for quantitative synthesis

11 studies included for quantitative synthesis

Supplementary Material S5: Risk of bias.

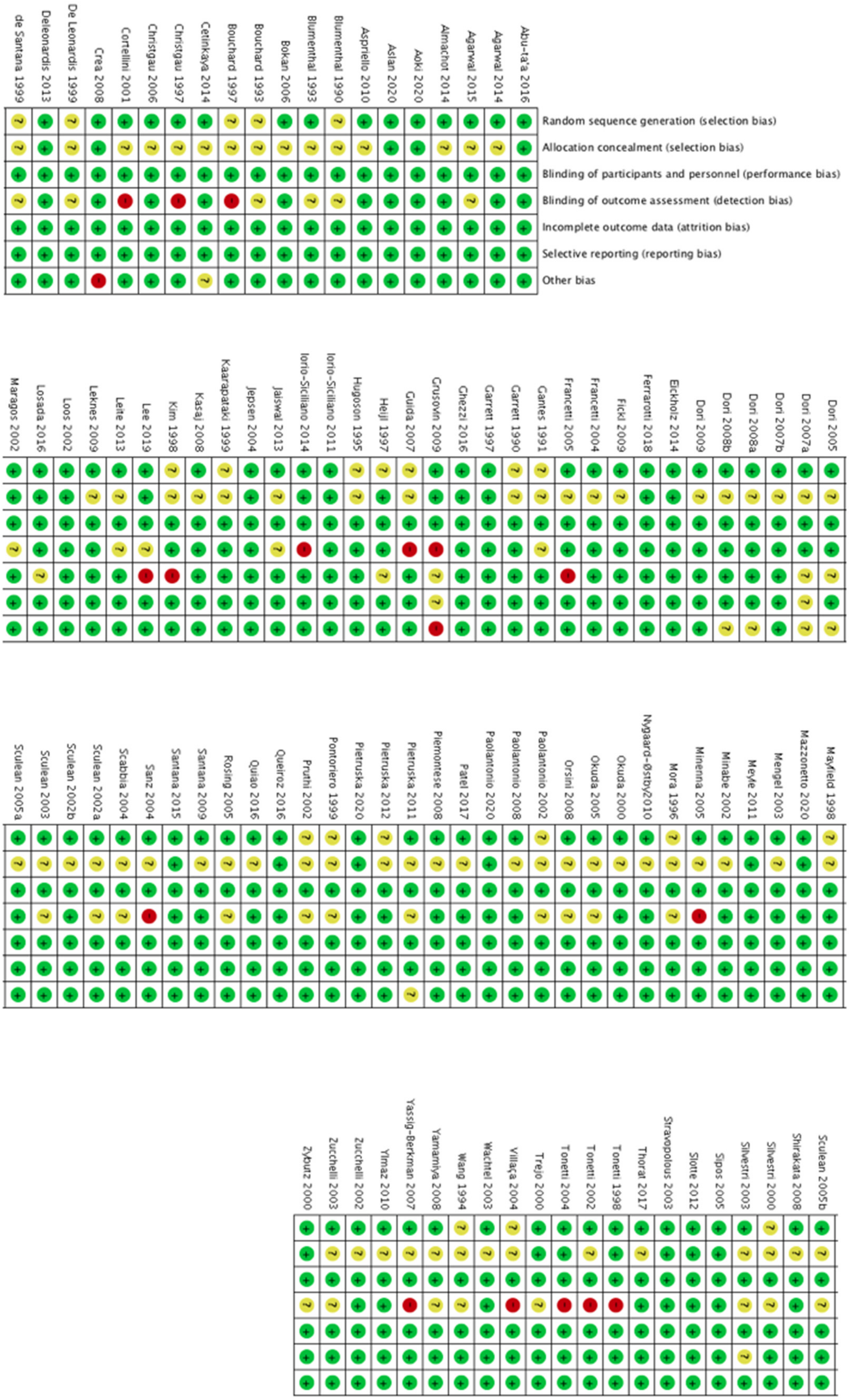

Supplementary Material S6: Forest plots of single-arm meta-analyses and meta-regression models using AB as factor in intrabony defects.

PPD reduction forest plot

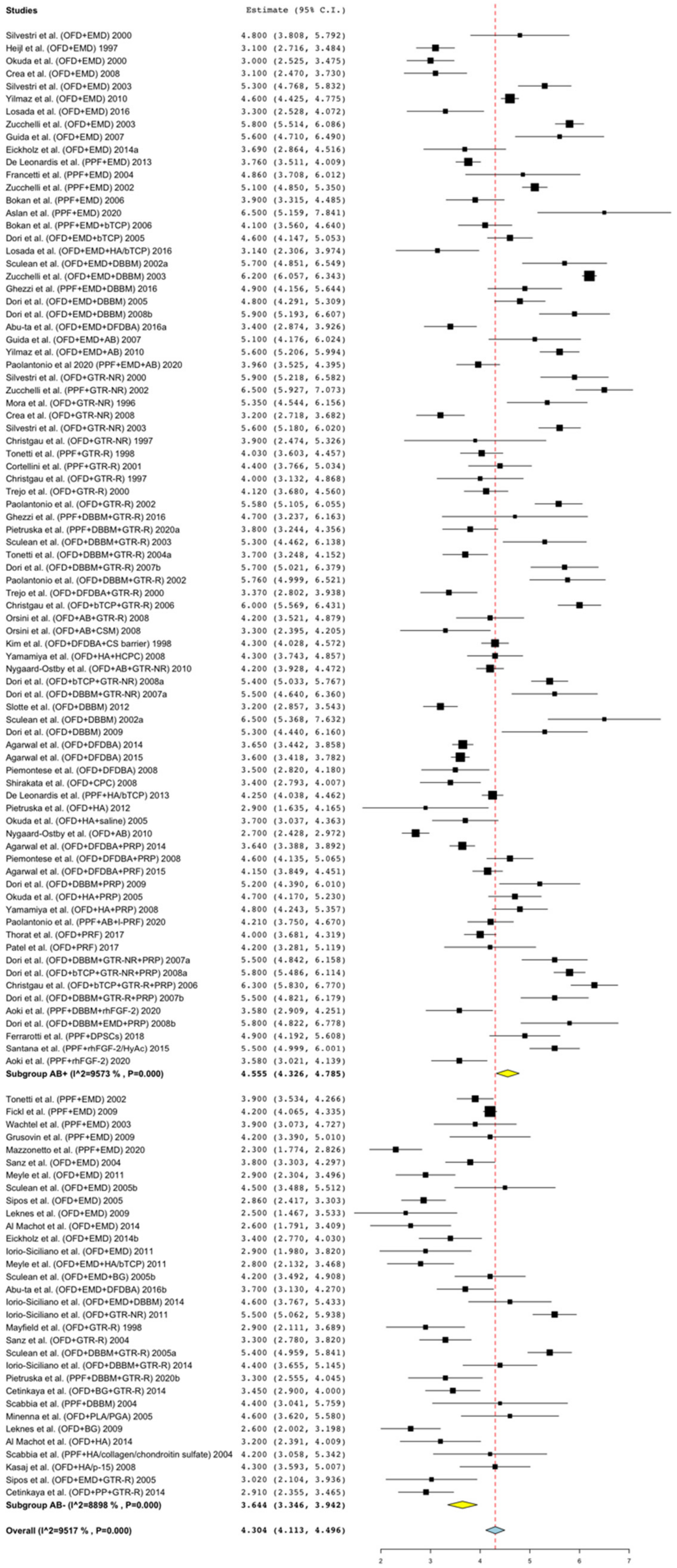

PPD reduction model results

Covariate	Level	Study arms	Coefficients	Lower bound	Upper bound	Std. error	*p*-Value
Intercept			4.553	4.346	4.759	0.105	< 0.001
AB	AB+	82					
	AB-	32	-0.908	-1.302	-0.514	0.201	< 0.001
Omnibus *p*-Value							0.000

PPD reduction adjusted means

Level	Study arms	Adjusted Means	Lower bound	Upper bound	Std. error
AB+	82	4.553	4.346	4.759	0.105
AB-	32	3.644	3.309	3.980	0.171

CAL gain forest plot

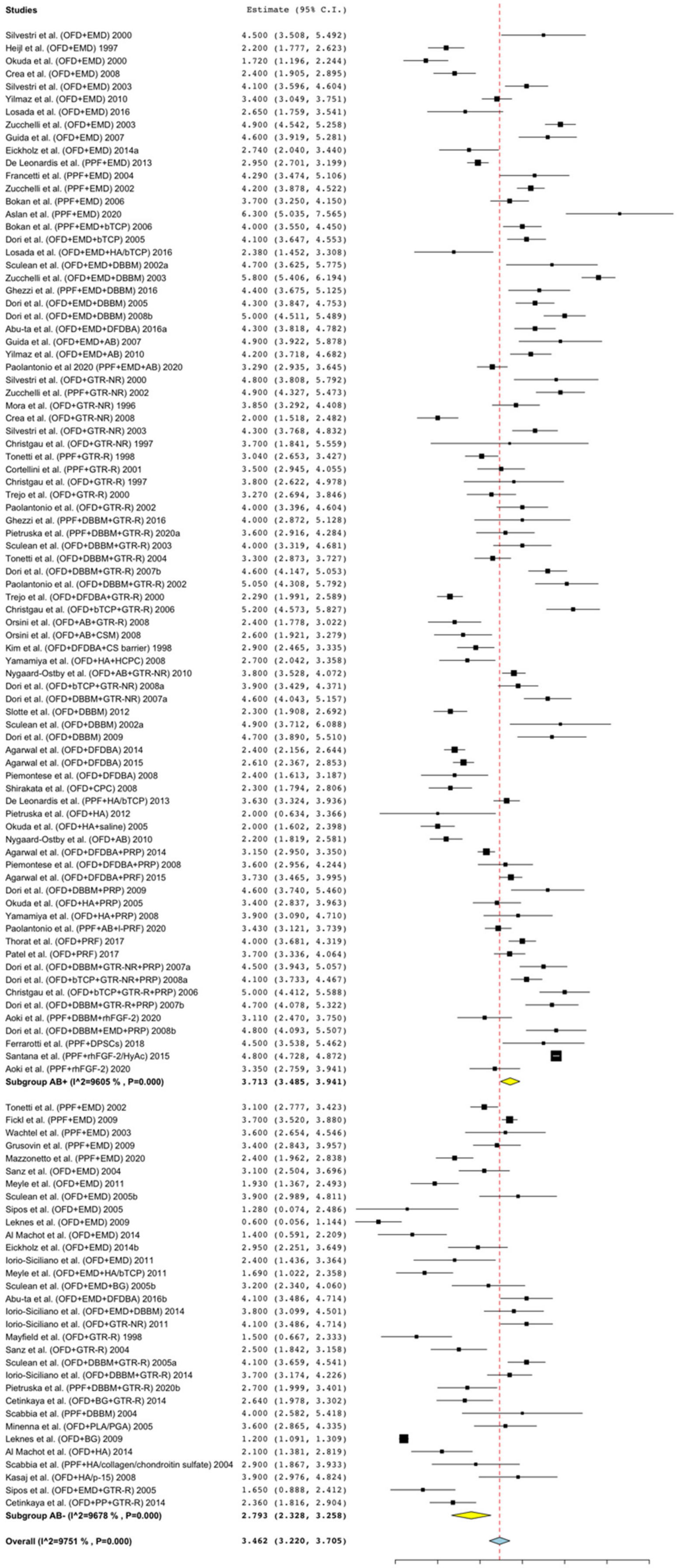

CAL gain model results

Covariate	Level	Study arms	Coefficients	Lower bound	Upper bound	Std. error	*p*-Value
Intercept			3.710	3.499	3.921	0.108	< 0.001
AB	AB+	82					
	AB-	32	-0.919	-1.322	-0.516	0.206	< 0.001
Omnibus *p*-Value							0.000

CAL gain adjusted means

Level	Study arms	Adjusted Means	Lower bound	Upper bound	Std. error
AB+	82	3.710	3.499	3.921	0.108
AB-	32	2.791	2.448	3.135	0.175

Bone gain forest plot

Studies	Estimate (95% C.I.)
Heijl et al. (OFD+EMD) 1997	2.600 (1.947, 3.253)
Crea et al. (OFD+EMD) 2008	2.700 (2.250, 3.150)
Yilmaz et al. (OFD+EMD) 2010	2.800 (2.449, 3.151)
Losada et al. (OFD+EMD) 2016	2.600 (1.770, 3.430)
Zucchelli et al. (OFD+EMD) 2003	4.300 (3.763, 4.837)
Guida et al. (OFD+EMD) 2007	4.300 (3.043, 5.557)
De Leonardis et al. (PPF+EMD) 2013	2.610 (2.445, 2.775)
Francetti et al. (PPF+EMD) 2004	3.440 (2.743, 4.137)
Losada et al. (OFD+EMD+HA/bTCP) 2016	2.710 (1.944, 3.476)
Zucchelli et al. (OFD+EMD+DBBM) 2003	5.300 (4.906, 5.694)
Guida et al. (OFD+EMD+AB) 2007	4.300 (3.593, 5.007)
Yilmaz et al. (OFD+EMD+AB) 2010	3.900 (3.462, 4.338)
Paolantonio et al 2020 (PPF+EMD+AB) 2020	2.670 (2.227, 3.113)
Mora et al. (OFD+GTR-NR) 1996	2.950 (2.144, 3.756)
Crea et al. (OFD+GTR-NR) 2008	2.700 (2.174, 3.226)
Trejo et al. (OFD+GTR-R) 2000	5.350 (3.826, 6.874)
Paolantonio et al. (OFD+GTR-R) 2002	3.820 (3.212, 4.428)
Pietruska et al. (PPF+DBBM+GTR-R) 2020a	3.200 (2.302, 4.098)
Paolantonio et al. (OFD+DBBM+GTR-R) 2002	5.230 (4.612, 5.848)
Trejo et al. (OFD+DFDBA+GTR-R) 2000	4.730 (4.152, 5.308)
Kim et al. (OFD+DFDBA+CS barrier) 1998	2.900 (2.139, 3.661)
Yamamiya et al. (OFD+HA+HCPC) 2008	3.200 (2.643, 3.757)
Nygaard-Ostby et al. (OFD+AB+GTR-NR) 2010	3.900 (3.465, 4.335)
Slotte et al. (OFD+DBBM) 2012	3.400 (2.273, 4.527)
Agarwal et al. (OFD+DFDBA) 2014	2.370 (2.182, 2.558)
Agarwal et al. (OFD+DFDBA) 2015	2.490 (2.261, 2.719)
Piemontese et al. (OFD+DFDBA) 2008	2.600 (1.956, 3.244)
Shirakata et al. (OFD+CPC) 2008	1.200 (0.795, 1.605)
De Leonardis et al. (PPF+HA/bTCP) 2013	3.350 (3.081, 3.619)
Pietruska et al. (OFD+HA) 2012	1.900 (1.141, 2.659)
Okuda et al. (OFD+HA+saline) 2005	2.700 (2.170, 3.230)
Nygaard-.stby et al. (OFD+AB) 2010	1.300 (0.811, 1.789)
Agarwal et al. (OFD+DFDBA+PRP) 2014	3.020 (2.820, 3.220)
Piemontese et al. (OFD+DFDBA+PRP) 2008	3.300 (2.763, 3.837)
Agarwal et al. (OFD+DFDBA+PRF) 2015	3.500 (3.260, 3.740)
Okuda et al. (OFD+HA+PRP) 2005	3.500 (3.003, 3.997)
Yamamiya et al. (OFD+HA+PRP) 2008	4.900 (4.293, 5.507)
Paolantonio et al. (PPF+AB+l-PRF) 2020	2.920 (2.623, 3.217)
Ferrarotti et al. (PPF+DPSCs) 2018	3.900 (3.141, 4.659)
**Subgroup AB+ (I^2=9371 % , P=0.000)**	**3.259 (2.992, 3.526)**
Fickl et al. (PPF+EMD) 2009	2.500 (2.320, 2.680)
Grusovin et al. (PPF+EMD) 2009	2.500 (1.893, 3.107)
Mazzonetto et al. (PPF+EMD) 2020	1.240 (0.740, 1.740)
Meyle et al. (OFD+EMD) 2011	2.810 (2.280, 3.340)
Sipos et al. (OFD+EMD) 2005	1.630 (0.915, 2.345)
Al Machot et al. (OFD+EMD) 2014	1.600 (1.060, 2.140)
Meyle et al. (OFD+EMD+HA/bTCP) 2011	2.650 (2.046, 3.254)
Mayfield et al. (OFD+GTR-R) 1998	0.600 (0.074, 1.126)
Pietruska et al. (PPF+DBBM+GTR-R) 2020b	2.500 (1.667, 3.333)
Cetinkaya et al. (OFD+BG+GTR-R) 2014	3.000 (2.125, 3.875)
Scabbia et al. (PPF+DBBM) 2004	3.100 (2.036, 4.164)
Al Machot et al. (OFD+HA) 2014	1.600 (1.060, 2.140)
Scabbia et al. (PPF+HA/collagen/chondroitin sulfate) 2004	2.500 (1.739, 3.261)
Sipos et al. (OFD+EMD+GTR-R) 2005	1.580 (0.445, 2.715)
Cetinkaya et al. (OFD+PP+GTR-R) 2014	3.450 (2.380, 4.520)
**Subgroup AB- (I^2=8506 % , P=0.000)**	**2.173 (1.790, 2.556)**
**Overall (I^2=9347 % , P=0.000)**	**2.971 (2.737, 3.205)**

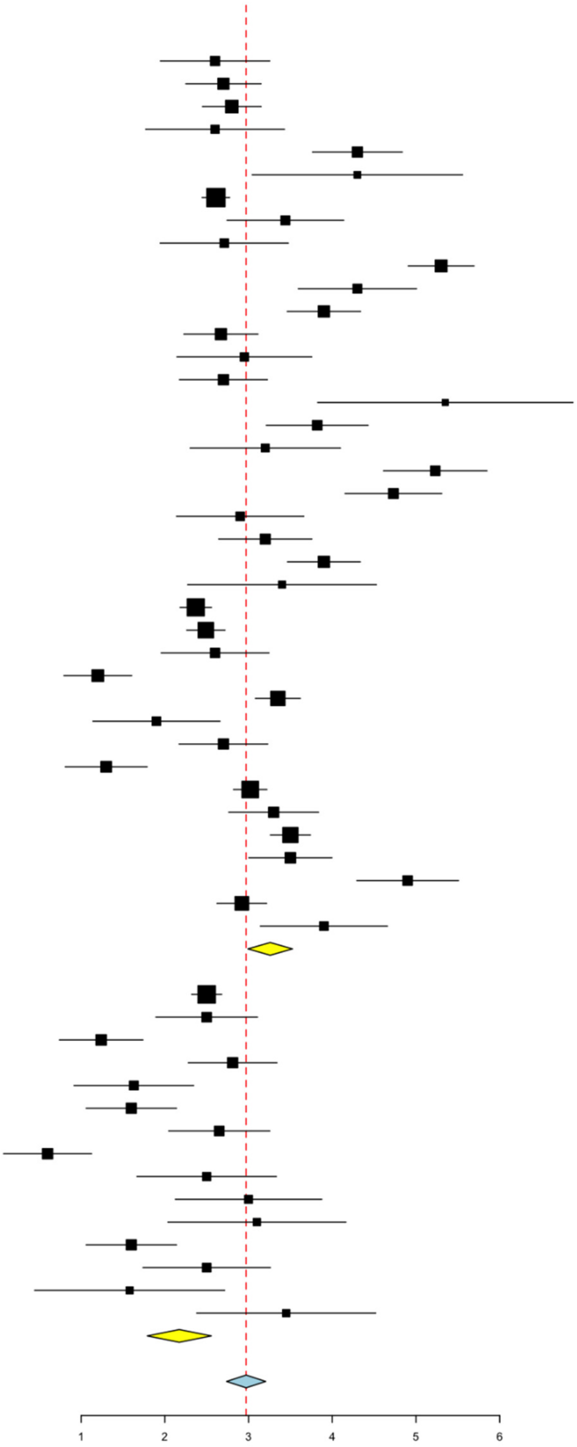

Bone gain model results

Covariate	Level	Study arms	Coefficients	Lower bound	Upper bound	Std. error	*p*-Value
Intercept			3.262	2.976	3.548	0.146	< 0.001
AB	AB+	39					
	AB-	15	-1.076	-1.627	-0.525	0.281	< 0.001
Omnibus *p*-Value							0.000

Bone gain adjusted means

Level	Study arms	Adjusted Means	Lower bound	Upper bound	Std. error
AB+	39	3.262	2.976	3.548	0.146
AB-	15	2.187	1.716	2.657	0.240

Supplementary Material S7: Meta-regression using AB as factor in intrabony defects for the EMD subgroup.

PPD reduction model results

Covariate	Level	Study arms	Coefficients	Lower bound	Upper bound	Std. error	*p*-Value
Intercept			4.372	3.919	4.825	0.231	< 0.001
AB	AB+	15					
	AB-	13	-0.984	-1.650	-0.318	0.340	0.004
Omnibus *p*-Value							0.004

PPD reduction adjusted means

Level	Study arms	Adjusted Means	Lower bound	Upper bound	Std. error
AB+	15	4.372	3.919	4.825	0.231
AB-	13	3.388	2.900	3.876	0.249

CAL gain model results

Covariate	Level	Study arms	Coefficients	Lower bound	Upper bound	Std. error	*p*-Value
Intercept			3.593	3.060	4.126	0.272	<0.001
AB	AB+	15					
	AB-	13	-0.980	-1.766	-0.193	0.401	0.015
Omnibus *p*-Value							0.015

CAL gain adjusted means

Level	Study arms	Adjusted Means	Lower bound	Upper bound	Std. error
AB+	15	3.593	3.060	4.126	0.272
AB-	13	2.613	2.035	3.192	0.295

Supplementary Material S8: Meta-regression using AB as factor in intrabony defects for the Graft +/- GTR-R subgroup.

PPD reduction model results

Covariate	Level	Study arms	Coefficients	Lower bound	Upper bound	Std. error	*p*-Value
Intercept			4.189	3.799	4.578	0.199	< 0.001
AB	AB+	23					
	AB-	10	-0.219	-0.941	0.504	0.369	0.553
Omnibus *p*-Value							0.553

PPD reduction adjusted means

Level	Study arms	Adjusted Means	Lower bound	Upper bound	Std. error
AB+	23	4.189	3.799	4.578	0.199
AB-	10	3.970	3.361	4.578	0.310

CAL gain model results

Covariate	Level	Study arms	Coefficients	Lower bound	Upper bound	Std. error	*p*-Value
Intercept			3.192	2.783	3.601	0.209	<0.001
AB	AB+	23					
	AB-	10	-0.154	-0.908	0.599	0.384	0.688
Omnibus *p*-Value							0.688

CAL gain adjusted means

Level	Study arms	Adjusted Means	Lower bound	Upper bound	Std. error
AB+	23	3.192	2.783	3.601	0.209
AB-	10	3.038	2.405	3.671	0.323

Supplementary Material S9: Meta-regression using AB type as factor in intrabony defects.

PPD reduction model results

Covariate	Level	Study arms	Coefficients	Lower bound	Upper bound	Std. error	*p*-Value
Intercept			4.717	4.456	4.978	0.133	< 0.001
AB Type	Penicillin	55					
	Tetracycline	17	-0.252	-0.792	0.288	0.276	0.360
Omnibus *p*-Value							0.360

PPD reduction adjusted means

Level	Study arms	Adjusted Means	Lower bound	Upper bound	Std. error
Penicillin	55	4.717	4.456	4.978	0.133
Tetracycline	17	4.465	3.992	4.937	0.241

CAL gain model results

Covariate	Level	Study arms	Coefficients	Lower bound	Upper bound	Std. error	*p*-Value
Intercept			3.924	3.688	4.161	0.121	<0.001
AB Type	Penicillin	55					
	Tetracycline	17	-0.240	-0.730	0.250	0.250	0.338
Omnibus *p*-Value							0.338

CAL gain adjusted means

Level	Study arms	Adjusted Means	Lower bound	Upper bound	Std. error
Penicillin	55	3.924	3.688	4.161	0.121
Tetracycline	17	3.685	3.255	4.114	0.219

Supplementary Material S10: Meta-regression using AB type as factor in furcation defects.

PPD reduction model results

Covariate	Level	Study arms	Coefficients	Lower bound	Upper bound	Std. error	*p*-Value
Intercept			1.889	1.427	2.350	0.235	< 0.001
AB Type	Penicillin	12					
	Tetracycline	9	0.341	-0.362	1.045	0.359	0.342
Omnibus *p*-Value							0.342

PPD reduction adjusted means

Level	Study arms	Adjusted Means	Lower bound	Upper bound	Std. error
Penicillin	12	1.889	1.427	2.350	0.235
Tetracycline	9	2.230	1.699	2.761	0.271

CAL gain model results

Covariate	Level	Study arms	Coefficients	Lower bound	Upper bound	Std. error	*p*-Value
Intercept			1.304	0.852	1.757	0.231	<0.001
AB Type	Penicillin	10					
	Tetracycline	9	0.313	-0.338	0.964	0.332	0.346
Omnibus *p*-Value							0.346

CAL gain adjusted means

Level	Study arms	Adjusted Means	Lower bound	Upper bound	Std. error
Penicillin	10	1.304	0.852	1.757	0.231
Tetracycline	9	1.617	1.149	2.086	0.239
